# Comparative Transcriptomics Reveals Distinct Adaptation Mechanisms for Degradation of *n*-Alkane and Branched Alkane in the Salt-Tolerant Bacterium *Dietzia* sp. CN-3

**DOI:** 10.3390/microorganisms13092206

**Published:** 2025-09-20

**Authors:** Weiwei Chen, Jiawei Sun, Xin Zhang, Jiawen Zhang, Yuan Wang, Shiwei Cheng

**Affiliations:** 1 School of Life Sciences, Ludong University, Yantai 264025, China; wwchen@ldu.edu.cn (W.C.); jwsun163@163.com (J.S.); 15192081793@163.com (X.Z.); jiawen66688@163.com (J.Z.); 2 Yantai Institute of Coastal Zone Research, Chinese Academy of Sciences, Yantai 264003, China; yuanwang@yic.ac.cn

**Keywords:** comparative transcriptomics, *n*-alkane, branched alkane, *Dietzia*

## Abstract

Although hydrocarbon-degrading bacteria utilize a wide range of alkanes, the global metabolic features and regulatory mechanisms governing their growth on alkanes with different chain lengths remain incompletely elucidated. In this study, we analyzed the comparative transcriptomics of a salt-tolerant bacterium, *Dietzia* sp. CN-3, to investigate molecular adaptations and metabolic processes when grown on *n*-hexadecane (C_16_), branched alkane (pristane), and glucose. A total of 1766 differentially expressed genes (DEGs) were identified in the C_16_ group compared to the glucose control, with 1024 (58.0%) being upregulated and 742 (42.0%) being downregulated. Notably, the pristane group exhibited 1542 DEGs, of which 488 (31.6%) were upregulated and 1054 (68.4%) were downregulated. Our results demonstrate that C_16_ and pristane induced common genes of alkane hydroxylation in the core alkane degradation pathway, while eliciting distinct transcriptional patterns of genes involved in lipid metabolism, energy metabolism, metal ion transportation, cell surface composition biosynthesis, and transcription regulation. The findings reveal that CN-3 employs diverse metabolic strategies to adapt to alkanes with different chain lengths, displaying considerable metabolic plasticity. This study significantly enhances our understanding of molecular adaptation of bacteria to hydrocarbon-containing environments and may provide valuable information for further studies of petroleum hydrocarbon bioremediation.

## 1. Introduction

Crude oil spills, resulting from natural or anthropogenic activities, contribute to substantial environmental contamination and pose severe challenges to ecological sustainability [1,2,3]. Alkanes, as major constituents of crude oil, could also be produced by many living organisms such as plants, algae, bacteria, or animals. Their widespread presence in the environment is especially conspicuous in areas contaminated by crude oil. The biodegradation of alkanes by microorganisms not only represents a feasible, economical, and eco-friendly remediation strategy but also underscores significant ecological and biotechnological value, particularly regarding oil spill cleanup, where it contributes to detoxification and ecosystem recovery [4,5,6]. Based on structural differences, alkanes can be classified as linear alkanes (*n*-alkanes), branched alkanes (*iso*-alkanes), or cycloalkanes. In comparison to linear alkanes, branched alkanes demonstrate higher environmental persistence, increased toxicity, and potential for biomagnification. These characteristics render them effective as diagnostic biomarkers for assessing the progress and efficacy of petroleum biodegradation [7,8,9,10].

Aerobic alkane degradation efficiently breaks down alkanes using oxygen, serving as the primary rapid response to oil spills in surface environments. Conversely, anaerobic degradation operates in oxygen-free zones like deep sediments and groundwater, playing a key role in the long-term subsurface carbon cycle, slowly utilizing sulfate or nitrate as electron acceptors [4,7,8]. Aerobic alkane degradation starts with alkane transportation, followed by a series of bio-oxidation reactions, including alkane hydroxylation, dehydrogenation, and fatty acid catabolism, which involve multiple enzymes such as hydroxylases, oxygenases, dehydrogenases, etc. [2,9]. Significantly, alkane hydroxylases exhibit strict substrate specificity, catalyzing hydroxylation reactions exclusively for alkanes with specific chain lengths. An earlier study suggested that long-chain alkane monooxygenase (LadA), identified in *Geobacillus thermodenitrificans* NG80-2, was responsible for C_15_-C_36_ oxidization but was not involved in <C_14_ alkanes [10]. Long-chain alkane monooxygenase (AlmA) was related to >C_32_ degradation in *Acinetobacter* sp. DSM 17874, which was also reported in *Alcanivorax*, *Marinobacter*, and *Parvibaculum* [11,12,13,14]. In *A*. *hongdengensis* strain A-11-3, expression of the integral-membrane alkane monooxygenase AlkB was strongly induced by medium-chain *n*-alkanes C_12_-C_16_ while demonstrating no responsive regulation to long-chain *n*-alkane C_28_ [12]. Additionally, branched alkanes pristane and phytane induced the expression of AlkB1 and AlmA in *A. hongdengensis* A-11-3 and *A. dieselolei* B5 [13,15]. In *D*. sp. DQ12-45-1b, soluble cytochrome P450 enzyme CYP153 was active on medium-chain *n*-alkanes C_8_-C_10_, and AlkW alkane hydroxylase was involved in long-chain *n*-alkanes C_12_-C_32_, which may have synergistic effects on broad-spectrum alkane degradation [16].

Bacterial cells access hydrophobic alkanes through producing biosurfactants and/or forming biofilm, enhancing substrates solubilization, uptake, and enzymatic reactions [17,18,19,20]. For instance, the DQ12-45-1b strain differentially produces distinct classes of biosurfactants of glycolipids, phospholipids, and lipopeptides in response to different *n*-alkane chain lengths of C_16_, C_24_, and C_36_, respectively [17]. Furthermore, based on the microscale dynamics of hexadecane degradation by four bacterial strains (*P. aeruginosa* PAO1, IMP68, O-2-2, and DQ12-45-1b), a positive correlation was observed between biofilm thickness and the degradation efficiency of oil droplets, which is likely attributable to the elevated cellular density and the abundant extracellular polymeric substances present within the biofilm [18].

Furthermore, previous studies investigated alkane metabolism regulation mechanisms in several typical bacterial strains. A signal transduction network was established in the marine hydrocarbonoclastic bacterium *A. dieselolei* B5, incorporating the key factors responsible for alkane sensing, chemotaxis, signal transduction, alkane uptake, and pathway regulation, elucidating the upstream metabolic and regulatory mechanisms of alkane [20]. The outer membrane proteins OmpT1, OmpT2, and OmpT3 exhibit distinct substrate specificities, selectively facilitating the transport of C_28_-C_36_, C_16_-C_24_, and C_8_-C_12_, respectively. In *D.* sp. DQ12-45-1b, the *CYP153* gene is positively regulated by the AraC family regulator CypR when using medium-chain *n*-alkanes, whereas *alkW1* is repressed by a TetR family regulator AlkX in the presence of long-chain *n*-alkanes [21,22]. CrgA participated in the alkane degradation regulation of *Pseudomonas aeruginosa* SJTD-1 by inhibiting the transcription of the *alkB2* gene, and it may also be responsible for the transcriptional regulation of long-chain alkane hydroxylases genes of *almA2*, *ladA1*, and *ladA2* [23]. Although previous studies have reported specific aspects, the global metabolic signatures and transcriptomic adaptation strategies employed by alkane-degrading bacteria under different chain lengths remain poorly characterized.

Previous transcriptomic studies have investigated bacterial metabolic mechanisms for petroleum hydrocarbon degradation. It was reported that genes involved in extracellular polysaccharide generation, efflux pumps and porins, biofilm formation, siderophore production, and multiple transcriptional regulators were induced when *P*. *aeruginosa* ATCC 33988 was cultivated with fuel as the sole carbon source [24]. A transcriptome analysis of *Lysinibacillus fusiformis* strain 15-4 recently showed that genes related to transporter activity, oxidoreductase/dehydrogenase activity, signal transduction, transcriptional regulation, and fatty acid catabolism were highly induced when strain 15-4 was grown in petroleum-containing medium compared with LB medium [25]. Another study reported that in *D.* sp. DQ12-45-1b, *n*-hexadecane (C_16_) and *n*-octacosane (C_28_) induced common genes of alkane degradation, whereas transcriptional profiles of genes involved in lipid metabolism, energy metabolism, and metal ion transportation were quite different [9]. Although prior studies have characterized bacterial transcriptional responses to alkanes or petroleum, little is known about the metabolic differences and transcriptional regulatory networks of bacteria growing on alkane substrates with different chain lengths.

As a Gram-positive bacterium, *Dietzia* was originally classified to the genus *Rhodococcus* and subsequently redefined as a distinct genus through comprehensive taxonomic analyses [26]. Members of this genus display broad environmental distribution, including marine and terrestrial environments, oil reservoirs, host-associated niches, and even extreme habitats [27,28]. Notably, *Dietzia* exhibits functional complementarity with both petroleum-degrading genera (*Acinetobacter*, *Bacillus*, etc.) [28,29,30,31] and non-degrading microorganisms (e.g., *Pseudomonas*) [28], and it helps establish syntrophic consortia to enhance petroleum bioremediation efficiency. *D.* sp. CN-3 was previously isolated from the petroleum-contaminated marine sediment of Bohai Sea, China. It is a salt-tolerant bacterium and can utilize a wide range of *n*-alkanes (C_14_-C_31_), branched alkanes, aromatic compounds, and crude oil as sole carbon and energy sources [31]. In this study, we employed comparative transcriptomics to elucidate differential gene expression and pathway profiles in CN-3 when grown on *n*-hexadecane (C_16_) and branched alkane (pristane). Our integrated transcriptomic analyses reveal that CN-3 adopts chain length-dependent alkane transcriptional mechanisms, providing novel insights into the networks of alkane-biodegrading bacteria.

## 2. Materials and Methods

### 2.1. Culture Media and Growth Conditions

*D*. sp. CN-3, which was obtained from crude oil-contaminated sediment as previously described [31], was deposited in the China Center for Type Culture Collection (CCTCC) with preservation number of CCTCC M 2015537. For hydrocarbon degradation assays, CN-3 strains were pre-cultured in LB medium at 30 °C for 36 h. Cells were then harvested by centrifugation at 8000 revolutions per minute (rpm) for 10 min and subsequently washed three times with mineral salt medium to remove residual nutrients [30]. The cells were cultured in the mineral salt medium supplemented with either 0.1% (*v*/*v*) *n*-hexadecane (C_16_), 0.1% (*v*/*v*) pristane, or 0.5% (*w*/*v*) glucose as the sole carbon source. The glucose-containing culture served as the control, and all cultures were incubated at 30 °C with shaking at 180 rpm. Bacterial growth was monitored by measuring the optical density at 600 nm (OD_600_), and growth curves were plotted accordingly. The degradation rates of C_16_ and pristane by CN-3 were also detected by gas chromatography-mass spectrometry (GC-MS, 7890, Agilent, Santa Clara, CA, USA) according to our previous study [31].

### 2.2. Biosurfactant Production Detection

Due to the detection of emulsification activity and cell surface hydrophobicity, strain CN-3 was able to produce biosurfactants when using tetradecane, paraffin oil, and crude oil as sole carbon sources [30]. The linear alkane *n*-hexadecane (C_16_) and the branched alkane (pristane) were employed as model hydrocarbon substrates to assess biosurfactant production performance. Specifically, following an incubation period of 7 days at 30 °C, cultures were centrifuged (10,000 rpm, 10 min) to separate cell-free supernatants and cell pellets. These fractions were applied severally in emulsification activity and cell surface hydrophobicity (CSH) assays to evaluate biosurfactant production as previously described [32,33].

### 2.3. Total RNA Extraction

Cells were harvested at the mid-exponential growth phase following cultivation on C_16_, glucose, or pristane as the sole carbon source, corresponding to incubation periods of 1 day, 2 days, and 14 days, respectively. All experiments were conducted with three independent biological replicates. Specifically, the bacterial pellets were harvested by centrifugation (8000 rpm) at 4 °C for 10 min, quickly frozen in liquid nitrogen for 15 min, and then sent to Shanghai Majorbio Bio-pharm Technology Co., Ltd. (Shanghai, China), for prokaryotic transcriptome sequencing. Total RNA was extracted from bacterial cultures using the RNAprep Pure Kit (TIANGEN, Beijing, China). The concentration and purity of RNA were quantified using a NanoDrop 2000 spectrophotometer (Thermo Fisher Scientific, Waltham, MA, USA). RNA integrity was evaluated using 1% agarose gel electrophoresis, and the RNA Integrity Number (RIN) was further determined with an Agilent 2100 Bioanalyzer (Agilent Technologies, Santa Clara, CA, USA).

### 2.4. cDNA Library Construction and Transcriptome Sequencing

Following ribosomal RNA (rRNA) depletion from total RNA extracts using the RiboCop rRNA Depletion Kit for Mixed Bacterial Samples (Lexogen, Billerica, MA, USA), mRNA from all samples was randomly broken into approximately 200 bp fragments by adding fragmentation buffer. Double-stranded cDNA was synthesized with random hexamer primers (Illumina). During second-strand cDNA synthesis, dUTP was incorporated as a substitute for dTTP. Subsequently, the synthesized cDNA underwent end-repair, phosphorylation, and ‘A’ base addition according to the standard Illumina library construction protocol. Finally, paired-end sequencing was conducted on an Illumina Novaseq 6000 platform using an Illumina Stranded mRNA Prep Ligation Kit (Illumina, San Diego, CA, USA) at Majorbio (Shanghai, China).

### 2.5. Sequence Assembly and Mapping

Following base calling, the raw reads were subjected to adapter and quality trimming using a combination of SeqPrep (v1.4) and Sickle (v1.33) to produce clean data for downstream analysis. Clean reads were obtained by removing low-quality sequences, reads with more than 10% of N bases, adaptor sequences, and small fragments with lengths less than 25 bp post mass pruning. The clean reads were mapped to the reference genome of the CN-3 strain by Bowtie2 (v2.5.2). The raw transcriptome sequencing data were deposited in the NCBI Sequence Read Archive (SRA) under accession number PRJNA1310299.

### 2.6. Expression Analysis and Functional Annotation

Gene expression quantification was performed using RNA-Seq with Expectation-Maximization (RSEM) software (v1.3.3). Expression levels were normalized using transcripts per million mapped reads (TPM) to enable comparative analysis of differentially expressed genes across samples. Unigene expression levels between the samples were analyzed using DESeq2 software (v1.48.2). The statistical significance of the obtained *p*-values was adjusted using the Benjamini-Hochberg (BH) approach to control the false discovery rate (FDR) in multiple comparisons. Differentially expressed genes (DEGs) were identified using a threshold of FDR ≤ 0.05 and an absolute value of log_2_(fold change) ≥ 0 [34]. Positive and negative log_2_(fold change) values represent gene upregulation and downregulation, respectively.

The DEGs were annotated to the Gene Ontology (GO, 20250516, http://geneontology.org/, accessed on 16 May 2025) database, Clusters of Orthologous Groups (COG, 20250516 http://www.ncbi.nlm.nih.gov/COG/, accessed on 16 May 2025) database, and Kyoto Encyclopedia of Genes and Genomes (KEGG, 20250516, http://www.genome.jp/tools/kaas/, accessed on 16 May 2025) database for functional annotation analysis and enriched by GO and KEGG pathways. In addition, the DEGs were aligned to the publicly available non-redundant (nr) database in NCBI (20250516, http://www.ncbi.nlm.nih.gov/, accessed on 16 May 2025) and Swiss-Prot protein databases with similarity set at >30% and an E-value ≤ 10^−5^.

### 2.7. Real-Time Quantitative PCR Validation

To validate the transcriptomic data, six genes of *alkB* (Die3_GM000017), *CYP153* (Die3_GM000105), *ADH* (Die3_GM001258, *ALDH* (Die3_GM000266, *fadD* (Die3_GM002355), and TetR (Die3_GM000016) and a reference gene (16S rRNA) were selected for real-time quantitative PCR (RT-qPCR) analysis. Total RNA extraction and RNA integrity assessment were performed as described above. The primers (Supplementary Materials, Table S1) were designed using Primer Premier 5.0 software and synthesized by Sangon Biotech Co., Ltd. (Shanghai, China). cDNA was synthesized using the PrimeScript FAST RT reagent kit with gDNA Eraser (Takara, Kusatsu, Japan) according to the instructions. RT-qPCR was performed using an ABI Prism 7500 Fast Real-Time PCR System (Applied Biosystems, Foster City, CA, USA). Reactions were carried out in a total volume of 20 µL containing 2X Universal SYBR Green Fast qPCR Mix (ABclonal, Wuhan, China) according to the manufacturer’s instructions. The thermal cycling protocol was initiated with a denaturation step at 95 °C for 3 min, followed by 43 cycles of 95 °C for 5 s and 60 °C for 32 s. Product specificity was evaluated using a melting curve. The threshold cycle values (Ct) were obtained from amplification curves, and relative differences in the gene expression fold changes were calculated through the 2^−ΔΔC^_T_ method [35]. All samples were analyzed in triplicate using a 96-well plate format, with three independent biological replicates per experimental group.

### 2.8. Construction of ΔalkB and ΔCYP153 Mutants

Based on the homologous recombination method, mutants of Δ*alkB* and Δ*CYP153* in CN-3 were constructed. Taking the *alkB* gene as an example, the homologous fragment (700 bp) was amplified from the genomic DNA with primers Dis-A-F/Dis-A-R (Supplementary Materials, Table S2). After digesting with XmaI and HindIII, the homologous fragment was ligated into pK18 to yield the suicide plasmid pK18-*alkB*. The plasmid was then electroporated (2200 V, 200 Ω) into competent cells of CN-3 using an electroporator (Gene Pulser Xcell Electroporation System, Bio-Rad, Hercules, CA, USA). The kanamycin-resistant clones were verified by PCR amplification and sequencing using primers Ver-A-F/Ver-A-R (Supplementary Materials, Table S2). The disruption mutant of Δ*alkB* was successfully constructed through correct integration of the homologous fragment into the chromosome, and similar procedures were used to construct the disruption mutant of Δ*CYP153*. The abilities of mutant strains to utilize *n*-alkane (C_16_) and branched alkane (pristane) were determined by monitoring cell growth (OD_600_) as described previously.

### 2.9. Statistical Analysis

Data are expressed as the mean ± standard deviation (SD) of three independent replicates. Statistical analyses were performed using IBM SPSS statistics 22. Significant differences among groups in growth conditions, biosurfactant production, and RT-qPCR validation results were assessed by one-way analysis of variance (ANOVA). Differences were considered statistically significant at *p* < 0.05.

## 3. Results and Discussion

### 3.1. Growth Characteristics, Degradation Rate, and Biosurfactant Production

Glucose, C_16_, or pristane was selected as a model substrate to evaluate the growth characteristics and biosurfactant production of strain CN-3. Notably, CN-3 exhibited superior growth on C_16_ compared to glucose, achieving peak biomass accumulation at 36 h. Particularly, according to the GC-MS measurement, the degradation rate of C_16_ by CN-3 was 97.2%. Although growth was slow, CN-3 could utilize pristane, reaching a maximum OD_600_ value of 0.712 (Supplementary Materials, Figure S1) and the highest degradation rate of 75.3%. Furthermore, when cultivated on C_16_, CN-3 displayed high emulsification activity (60.94 ± 3.32%) and CSH (76.51 ± 4.34%). In contrast, pristane-grown cultures showed high emulsifying activity (56.25 ± 3.64%) but significantly lower CSH (36.5 ± 4.64%). Bacteria always employ two key strategies to degrade hydrophobic hydrocarbons: secreting extracellular biosurfactants to increase hydrocarbon bioavailability and modulating CSH to facilitate hydrocarbon–cell interactions. These mechanisms may operate simultaneously or independently [17,32]. Considering the emulsification activity and CSH results, strain CN-3 appears to employ divergent substrate utilization strategies for *n*-hexadecane (C_16_) and pristane. Similarly, although *D. maris* As-13-3 produced di-rhamnolipid as a biosurfactant and exhibited high emulsification activity (E_24_ 55.88%) and strong CSH (61.2%) toward C_16_, it showed markedly reduced hydrophobicity (38.8%) in the presence of pristane [32].

### 3.2. General Features of Transcriptome in Three Carbon Sources

The transcriptomic profiles of strain CN-3 cultivated on *n*-alkane (C_16_), branched alkane (pristane), and glucose (control) were comprehensively analyzed through high-throughput RNA sequencing (RNA-Seq). We obtained a total of 20876008-39258372 clean reads from triplicate biological replicates for each growth condition. These reads accounted for genome coverage ranging from 72.58% to 98.3% (Supplementary Materials, Table S3). As shown in Figure S2 in the Supplementary Materials, three parallel samples displayed strong linear relationships based on TPM values.

The RNA-Seq results were further validated using RT-qPCR. Six DEGs were selected, and 16S rRNA was the reference gene. As shown in Figure S3, the relative expression of DEGs according to RT-qPCR was consistent with that observed with RNA-Seq (R^2^ = 0.8861/0.9109), thus reflecting the high quality of sequencing data.

**Figure 1:**
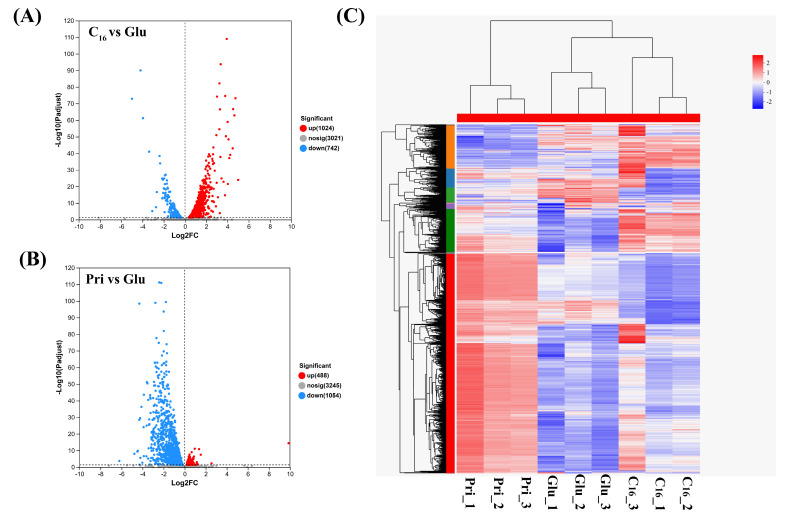
Global transcriptional profiling of DEGs in CN-3 cultured with C_16_, pristane (Pri), and glucose (Glu) as sole carbon sources. Volcano plots show differently expressed genes of CN-3 during growth on C_16_ versus glucose (C_16_ vs. Glu) (**A**), pristane versus glucose (Pri vs. Glu) (**B**). The horizontal axis is the log_2_ (fold change); the vertical axis is the FDR values. The plots in red, blue and gray display the significantly up-regulated, significantly down-regulated and not significantly changed genes, respectively. (**C**) Heat map analysis of transcript abundance in CN-3 cultured with C_16_, pristane (Pri), and glucose (Glu) as sole carbon sources. Blue, downregulated; red, upregulated. Transcript abundance was normalized and expressed based on TPM values.

Overall, there were 1766 DEGs when comparing the C_16_ group with the glucose control, wherein 1024 DEGs (58.0%) were upregulated and 742 DEGs (42.0%) were downregulated (Figure 1A). Additionally, of the 1542 differentially expressed genes (DEGs) identified in the pristane group compared to the glucose control, 488 (31.6%) were upregulated, while 1054 (68.4%) were downregulated (Figure 1B). However, only 17/132 DEGs were identified to be co-upregulated/downregulated in C_16_ versus glucose or pristane versus glucose, respectively (Supplementary Materials, Figure S4). We also found that 3259 DEGs and 1584 (48.6%)/1675 (51.4%) DEGs were up/downregulated in pristane versus C_16_, respectively. A heat map analysis of transcript abundance revealed carbon source-specific gene expression patterns, with clear segregation in glucose-, C_16_-, and pristane-grown cultures (Figure 1C). These results demonstrate distinct transcriptional responses to different alkane substrates of *n*-alkane (C_16_) and branched alkane (pristane) in strain CN-3.

### 3.3. Functional Classification

**Figure 2:**
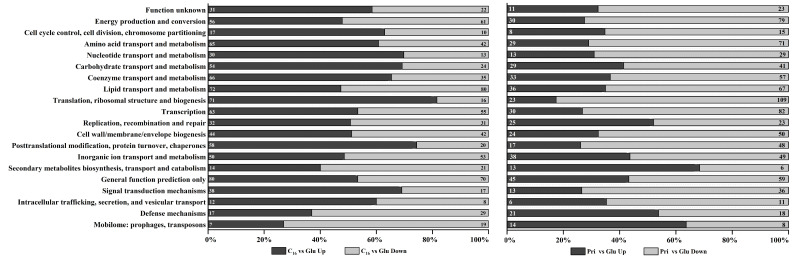
Differentially expressed genes (DEGs) identified from comparisons of *n*-hexadecane versus glucose (C_16_ vs. Glu) and pristane versus glucose (Pri vs. Glu) were functionally classified according to the Clusters of Orthologous Groups (COG) database. The vertical axis corresponds to COG functional categories, while the horizontal axis represents the percentage of DEGs assigned to each category. Upregulated and downregulated genes are indicated in dark gray and light gray, respectively. The numerical values displayed within the bars denote the count of enriched DEGs per category.

To investigate the transcriptional underpinnings of hydrocarbon degradation, differentially expressed genes (DEGs) were systematically annotated and functionally categorized using the COG database. As illustrated in Figure 2, DEGs from C_16_ versus glucose or pristane versus glucose were classified into 20 categories. Notably, within the functional category “Replication, recombination and repair”, the number of upregulated differentially expressed genes (DEGs) exceeded that of downregulated DEGs in both the hexadecane (C_16_) versus glucose and the pristane versus glucose comparisons. It was reported that the upregulation of related genes on “replication, recombination and repair” may enhance the survival ability of bacteria in petroleum hydrocarbon-polluted environments [9,25]. In the functional category of “Energy production and conversion”, a greater number of DEGs were downregulated compared to those upregulated in both experimental groups, which illustrated that CN-3 was in a lower energy state when using C_16_ and pristane as sole carbon sources than when using glucose. The functional annotation demonstrated that CN-3 had metabolic commonalities when grown on C_16_ and pristane. However, in an overwhelming majority of categories, such as “Amino acid transport and metabolism”, “Nucleotide transport and metabolism”, “Carbohydrate transport and metabolism”, “Translation, ribosomal structure, and biogenesis”, “Transcription”, “Secondary metabolites biosynthesis, transport and catabolism”, “Signal transduction mechanisms”, “Intracellular trafficking, secretion, and vesicular transport”, and “Defense mechanisms”, both the trend variation and the number of upregulated/downregulated DEGs differed significantly between these two comparable groups. Overall, functional classification of DEGs revealed distinct transcriptional patterns across multiple metabolic pathways when cells were grown on C_16_ or pristane despite some shared features, suggesting that CN-3 employs different metabolic strategies for *n*-alkane (C_16_) and branched alkane (pristane) utilization.

### 3.4. Core Alkane Degradation

#### 3.4.1. Alkane Uptake

The initial step in bacterial hydrocarbon assimilation involves cellular alkane uptake; however, the molecular mechanisms of uptake systems are not yet fully elucidated. The mechanism may differ due to the bacterial species, alkane length, and environmental physicochemical characteristics [22,36]. It was reported that biosurfactants contributed to increasing the uptake and assimilation of alkanes such as hexadecane in liquid cultures. Glycolipids and lipoproteins are known as two typical biosurfactants synthesized by hydrocarbon-degrading bacteria [17,25]. In this study, six genes encoding glycosyl transferases that may be responsible for lipopolysaccharide biosynthetic were identified, with four genes being upregulated during C_16_ growth and another two genes being upregulated under pristane conditions (Table 1). Furthermore, recent studies have demonstrated that several outer membrane proteins, including AlkL, FadL, AupA, and OmpT, play crucial roles in facilitating alkane uptake and transportation in Gram-negative bacteria [9,22,37]. It was also reported that in *A. borkumensis* SK2 and *P. aeruginosa* ATCC 33988, an outer membrane lipoprotein gene *blc* contributed to alkane assimilation and cell survival in hydrocarbon-rich environments [36,37,38,39]. Similarly, one membrane-anchored lipoprotein gene *blc* (Die3_GM000935), one ABC transporter permease gene (Die3_GM003229), and one adhesin gene *znuA* (Die3_GM000578), mediating active solute transport across the cytoplasmic membrane in Gram-positive bacteria, were upregulated under C_16_ or pristane conditions compared with glucose. Together, these genes represent promising yet unvalidated candidates; however, their precise functions and roles within the core alkane degradation mechanism of CN-3 remain to be confirmed through genetic approaches (e.g., knockout or heterologous expression).

#### 3.4.2. Alkane Oxidation

Aerobic alkane degradation is mediated through a series of oxidation reactions. Alkane hydroxylation, catalyzed by specialized alkane monooxygenases, is a critical and initial oxidation step that facilitates the incorporation of molecular oxygen into hydrocarbon substrates [22]. One alkane hydroxylase-rubredoxin fusion protein AlkB and one cytochrome P450 hydroxylase of the CYP153 encoding gene were identified in the genome of CN-3.

**Figure 3:**
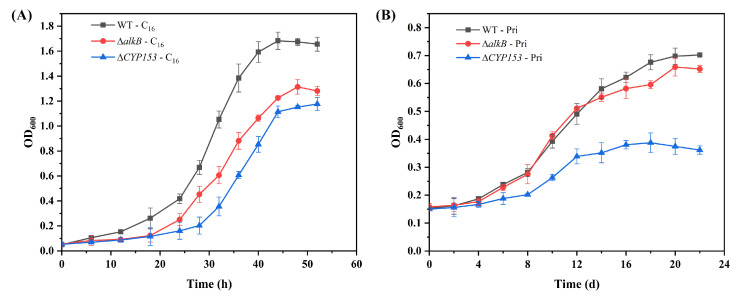
The growth curves of the wild-type CN-3 strain (WT) and Δ*alkB* and Δ*CYP153* mutant strains under C_16_ (**A**) and pristane (**B**) growth conditions. Error bars indicate the standard deviation of three biological replicates.

In general, AlkB and CYP153 encoding genes are reported to be involved in the oxidation of short-, medium-, and long-chain alkanes (C_5_-C_17_) [9,16]. In this study, *alkB* (Die3_GM000017) was significantly induced in the C_16_ versus glucose group (17.1-fold) but repressed in the pristane versus glucose group (0.98-fold). *CYP153* (Die3_GM000105) was significantly induced by C_16_ (26.9-fold) and pristane (2.2-fold) when compared with glucose. To investigate the potential essentiality of *alkB* and *CYP153* in the degradation of both C_16_ and pristane, we constructed targeted deletion mutants (Δ*alkB* and Δ*CYP153*) using homologous recombination. This approach allowed us to experimentally evaluate the phenotypic consequences of each gene disruption and directly test their hypothesized roles in alkane metabolism. As shown in Figure 3, the growth of Δ*alkB* and Δ*CYP153* mutants was slightly inhibited under C_16_ conditions. When cultivated on pristane, the Δ*alkB* mutant and wild-type strain CN-3 showed comparable growth, while the Δ*CYP153* mutant was significantly inhibited. These results are nearly consistent with the RT-qPCR detection results in our previous study showing that the *CYP153* gene was dramatically induced by C_14_, C_15_, C_16_, C_26_, and pristane, whereas the *alkB* gene was moderately induced by C_16_ and C_26_ and only weakly detected by C_14_, C_15_, and pristane [31].

In addition, the ferredoxin (Die3_GM000104) and ferredoxin reductase (Die3_GM000106) genes, located adjacent to *CYP153*, exhibit co-upregulated expression in both the C_16_ versus glucose and pristane versus glucose groups, which are always required as electron transfer systems for cytochrome P450 [16,40]. Based on the RNA-seq, RT-qPCR, and disruption mutant construction results, *alkB* and *CYP153* genes might have distinct roles in alkane hydroxylation. Specifically, *alkB* was more prominent in *n*-alkane (C_16_) utilization, and *CYP153* played crucial roles in *n*-alkane (C_16_) and branched alkane (pristane) degradation by CN-3, exhibiting certain discrepancies compared to previous reports [9,34,41]. For example, in the DQ12-45-1b strain, RNA-seq analysis revealed markedly higher induction of *CYP153* by C_16_ (21.6-fold) than by C_28_ (1.6-fold). Similarly, *alkW1* and *alkW2* expression was strongly upregulated by C_16_ (29- and 54-fold, respectively), compared to more moderate induction by C_28_ (14- and 7-fold), relative to the glucose control [9]. RT-qPCR analysis indicated that *alkB* and *CYP153* expression was strongly induced by C_14_, C_16_, and pristane (>20-fold) in *D. maris* As-13-3 [32]. Moreover, two Baeyer–Villiger monooxygenase-encoding genes (Die3_GM001585 and Die3_GM003194) were found in CN-3. Die3_GM003194 exhibited significant upregulation in both C_16_ and pristane conditions relative to glucose, suggesting potential subterminal oxidation of alkanes [41]. Together, most of the genes responsible for alkane hydroxylation in the core alkane degradation pathway were co-upregulated in CN-3 when grown on *n*-alkane (C_16_) and branched alkane (pristane).

The third step of alkane degradation involves oxidation of the primary alcohol to its corresponding aldehyde, catalyzed by alcohol dehydrogenases (ADHs), followed by further oxidation to a fatty acid, mediated by aldehyde dehydrogenases (ALDHs) [9,25]. Seven ADH homologues and seven ALDH homologues in CN-3 genome were identified through multiple sequence alignment or phylogenetic analysis of ADH or ALDH homologues from alkane-degrading bacteria (Supplementary Materials, Figures S5 and S6). Particularly, five ADH homologues (Die3_GM000170, Die3_GM001258, Die3_GM001307, Die3_GM002689, and Die3_GM002991) were induced in C_16_ versus glucose, but they were all repressed in pristane versus glucose. Two ADH homologues (Die3_GM001956 and Die3_GM001307) were repressed in C_16_ versus glucose but induced in pristane versus glucose (Table 1). Similarly, six ALDH coding genes (Die3_GM000037, Die3_GM000266, Die3_GM002359, Die3_GM002915, Die3_GM000896, and Die3_GM002352) showed different transcriptional patterns between the C_16_ versus glucose group and the pristane versus glucose group. This expression pattern is likely attributable to variations in the transcriptional regulation of genes involved in distinct alkane metabolic pathways [22].

Alkane uptake, alkane hydroxylation, and dehydrogenation reactions are the core alkane degradation processes. Based on the transcriptional profiles of key genes, despite some co-upregulated/downregulated features, the core alkane degradation pathway in CN-3 exhibits distinct substrate specificity when grown on *n*-alkane C_16_ and branched alkane pristane.

### 3.5. Lipid Metabolism

**Figure 4:**
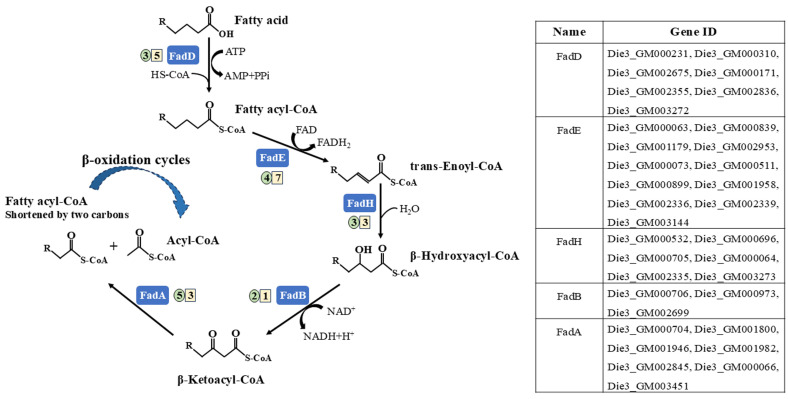
Transcriptional profile of fatty acid β-oxidation genes in CN-3 grown on C_16_ and pristane conditions. The black filled rectangles represent the enzymes of β-oxidation (FadD, fatty-acyl CoA synthase; FadE, acyl-CoA dehydrogenase; FadH, enoyl-CoA hydratase; FadB, 3-hydroxybutyryl-CoA dehydrogenase; and FadA, acetyl-CoA acyltransferase). The values in circles and squares represent the induced DEGs numbers in C_16_ versus glucose and pristane versus glucose.

Alkane oxidation generates significant amounts of fatty acids, which subsequently undergo catabolic degradation via the β-oxidation pathway [2,14]. To investigate the lipid metabolism of strain CN-3, the transcriptional expression of genes involved in fatty acid degradation was analyzed. Generally, fatty acid degradation mainly consists of five steps, namely fatty acid activation, dehydrogenation, hydration, re-dehydrogenation, and sulfonation, which are mediated by *fadD* (encoding fatty acyl-CoA synthase), *fadE* (encoding acyl-CoA dehydrogenase), *fadH* (encoding enoyl-CoA hydratase), *fadB* (encoding 3-hydroxybutyryl-CoA dehydrogenase), and *fadA* (encoding acetyl-CoA acyltransferase), respectively [9,25,42]. Comparative transcriptomic analysis revealed that C_16_ and pristane induced distinct transcriptional profiles, with several common induced expressed genes when compared to the glucose control (Supplementary Materials, Table S4): *fadD* (Die3_GM002675), *fadE* (Die3_GM000063), *fadB* (Die3_GM000973), and *fadA* (Die3_GM001946). Furthermore, in the initial fatty acid β-oxidation steps of activation and dehydrogenation, the numbers of *fadD* and *fadE* homologous genes were significantly higher in cells cultured on pristane than in those on C_16_ (Figure 4). However, during the downstream steps of β-oxidation, two *fadB* homologs genes (Die3_GM000705 and Die3_GM000973) exhibited significant upregulation in C_16_ versus pristane. In the final step of fatty acid oxidation, five *fadA* homologous genes were significantly induced in C_16_ versus glucose, whereas only three *fadA* genes were induced by pristane.

In Gram-negative bacteria of *Acinetobacter oleivorans* DR1, *fadD*, *fadE*, *fadB*, and *fadH* were induced by C_16_ versus succinate [43]. In Gram-positive bacteria of *Lysinibacillus fusiformis* 15-4, diverse fatty acid oxidation genes such as *fadD*, *fadB*, and *fadA* were upregulated, implying that the strain had a robust capacity for fatty acid catabolism when grown on petroleum [25]. Studies have reported that *D*. species possess a considerable number of enriched genes related to lipid metabolism in the genomes, indicating a distinctive genomic signature that distinguishes *D*. from other species [44]. The comparative transcriptional analysis performed in our study demonstrated that C_16_ and pristane induce distinct sets of fatty acid β-oxidation-related genes in CN-3, suggesting different metabolic fates of fatty acids under two alkane chain lengths. C_16_ predominantly induced genes associated with the downstream reactions of fatty acid oxidation, which facilitates complete fatty acid degradation into acetyl-CoA—a critical metabolic product that supports rapid bacterial growth under C_16_ conditions. In contrast, pristane preferentially activates genes involved in the upstream catalytic steps of fatty acid oxidation, which is beneficial for promoting the accumulation of intermediate metabolites rather than full oxidation.

### 3.6. Energy Metabolism

The tricarboxylic acid (TCA) cycle constitutes a central metabolic pathway responsible for energy generation in aerobic bacteria [2,9]. To investigate the differential energy metabolism of CN-3, we analyzed transcriptional differences in key genes associated with the TCA cycle. In the group of C_16_ versus glucose, most genes related to the TCA cycle were significantly induced, but their transcriptional activity was uniformly suppressed when grown on pristane compared to on glucose (Table 2). These results indicate a reduced energetic status in CN-3 during growth on pristane relative to C_16_, consistent with its suboptimal growth on this branched-chain substrate. Additionally, as shown in Table S5, the homologous genes associated with the oxidative phosphorylation machinery—specifically those encoding ATP synthesis, cytochrome d/b biosynthesis, cytochrome c biosynthesis, purine biosynthesis, pyrimidine biosynthesis, and de novo synthesis—exhibited significantly stronger repression in CN-3 when cultured on pristane compared to C_16_. In contrast, NADH dehydrogenases (ubiquinone) (complex **I**), multi-subunit enzymes encoded by the gene clusters of *nuoABCDEFGHIJKLMN*, were obviously upregulated when grown on pristane compared to C_16_. Previous studies demonstrated that the main function of complex I was to catalyze the oxidation of NADH, transfer electrons to the respiratory chain, and simultaneously couple proton transmembrane transport to drive ATP synthesis [45]. During the transfer of two electrons from NADH to menaquinone via flavin mononucleotide (FMN) and iron-sulfur (Fe-S) clusters, complex **I** catalyzed the translocation of four protons across the cytoplasmic membrane. Therefore, although the CN-3 strain was in a low-energy state under pristane growth conditions, the upregulation of complex I probably contributed to maintaining membrane potential and electron transfer efficiency [46,47]. Notably, the upregulation of complex **I** might be attributed to the large number of NADH-dependent dehydrogenases such as ADHs, ALDHs, and alkane hydroxylase AlkB that were induced under pristane growth conditions. This was conducive to the bacteria sustaining the oxidation–reduction reactions during the low-energy growth state, which might be an adaptive evolutionary strategy for strain CN-3.

The glyoxylate pathway plays a critical role in bacterial energy metabolism when grown on hydrocarbons [48,49]. In this study, the transcriptional expression of two genes involved in the glyoxylate pathway was analyzed: *aceA* (encoding isocitrate lyase) and *aceB* (encoding malate synthase) were both significantly upregulated when grown on C_16_ compared to on glucose, but their expression levels were significantly downregulated when grown on pristane versus glucose, indicating that the glyoxylate pathway had a different influence on C_16_ and pristane degradation. Similarly, the growth characteristics of the Δ*aceA* mutant in *D*. sp. DQ12-45-1b were completely repressed when grown on C_16_, while still growing weakly under the C_28_ condition, implying that the glyoxylate pathway was essential for C_16_ degradation but dispensable for C_28_ degradation [2,49]. The reason for these results might be the oxidative stress and its associated metal toxicity, but further experimental validation is needed.

### 3.7. Metal Ions Transportation

**Figure 5:**
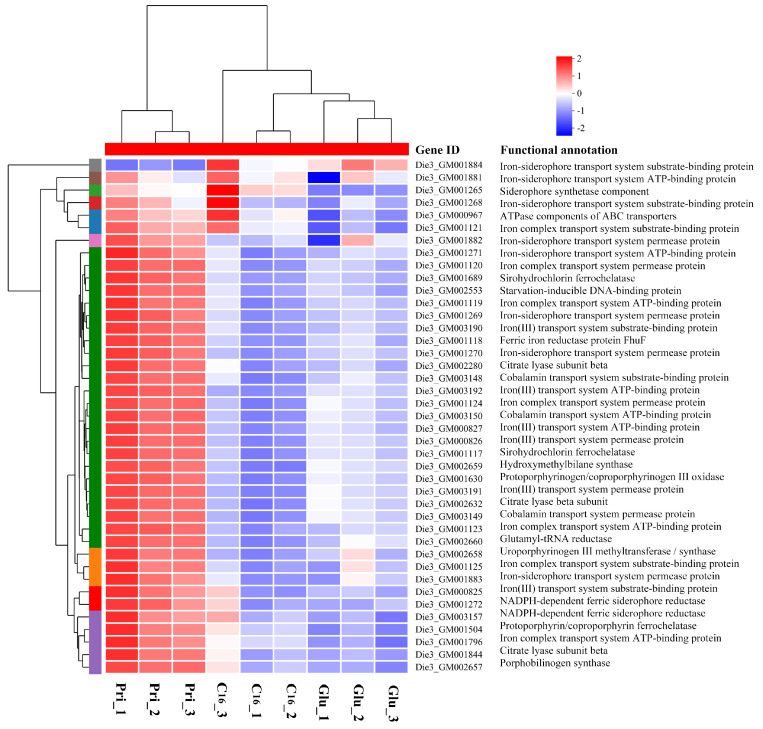
Heatmap depicting the transcription of iron transport and siderophore biosynthesis genes in CN-3 cultivated with C_16_, pristane, or glucose. Expression values are normalized TPM averages.

Iron plays critical roles in numerous biological processes for living cells; it also acts as an electron carrier in iron–sulfur proteins. To elucidate metal ion transport mechanisms, we identified putative iron transporter genes and characterized their transcriptional regulation patterns. Intriguingly, 29 genes related to iron transport and siderophore biosynthesis in CN-3 were upregulated when grown on C_16_ versus glucose, while these genes were nearly downregulated when grown on pristane (Figure 5). Similarly, comparative transcriptomic analysis revealed that C_16_ and pristane induced distinct transcriptional profiles related to transportation of Mn^2+^, Zn^2+^, Cu^2+^, and Co^2+^, with several common induced expressed genes including *MntR* (Die3_GM001396), *cobB-cbiA* (Die3_GM001077), and *cobL-cbiET* (Die3_GM001080) when compared to the glucose control (Supplementary Materials, Table S6). Furthermore, the *furA* gene (Die3_GM000457) encoding a negative regulator of iron uptake was significantly repressed under both C_16_ and pristane growth conditions, indicating that it promoted iron absorption in these alkanes. In addition, the cation efflux pump encoding gene (Die3_GM002064) was induced when grown on pristane.

Iron is an essential cofactor for a broad spectrum of proteins and enzymes involved in hydrocarbon oxidation, facilitating electron transfer in key metabolic pathways. These include alkane monooxygenases (e.g., AlkB), dehydrogenases, ferredoxins, thioredoxins, thioredoxin reductases, and ferredoxin-NADP reductase [4,25]. Genes involved in iron uptake were specifically induced in *A. borkumensis* SK2 when grown on alkanes versus pyruvate [38]. Furthermore, in *D.* sp. DQ12-45-1b, 52 iron transport genes were markedly upregulated when grown on C_16_ but downregulated on C_28_ [9]. However, in *P. aeruginosa* ATCC 33988, membrane transporters were downregulated when grown on jet fuel [36]. These findings imply that distinct molecular machinery adapted to hydrocarbon degradation may have evolved in different bacterial strains. In this study, most genes associated with the transport of iron and other metal ions were induced in C_16_ versus glucose, which is consistent with prior studies. In contrast, these genes exhibited significant repression when grown on pristane, likely attributable to reduced metabolic activity and diminished biomass production in the CN-3 strain. The CN-3 strain exhibited slower metabolic activity under pristane growth conditions. We proposed that the differential expression pattern of metal ion transporter genes not only mitigates cell toxicity caused by ion accumulation but also reduces energy expenditure for the biosynthesis of related proteins during pristane utilization [50]. Furthermore, the regulatory mechanisms of metal ion transport in CN-3 when grown on C_16_ or pristane remain incompletely understood.

### 3.8. Biosynthesis of Cell Surface Compositions

The limited aqueous solubility of hydrocarbons drives bacterial cells to increase CSH, a critical adaptive strategy enabling direct substrate interaction and enhanced biodegradation efficiency [32,51]. Transcriptional analysis of key genes was performed to investigate the contribution of surface protein, poly-L-glutamine (PLG), and lipids to bacterial CSH enhancement [52]. Notably, the biosynthesis of surface proteins involves secretion (Sec) system-mediated translocation of unfolded precursors, followed by their extracellular folding into mature proteins [42,53]. As important parts of Sec systems, SecD and SecF play indispensable roles in sustaining optimal protein translocation efficiency across the cytoplasmic membrane. Compared with the glucose control, *secF* (Die3_GM001600) and *secD* (Die3_GM001601) genes were significantly upregulated when cells used C_16_ as sole carbon source, but there were no significant transcriptional differences when cells were under the pristane cultivation condition (Supplementary Materials, Table S7). Moreover, *groES* (Die3_GM000756) and *groEL* (Die3_GM000756), encoding chaperones for protein folding [54], were upregulated in the C_16_ versus glucose group but downregulated in the pristane versus glucose group. Glutamine synthetase genes play crucial roles in PLG biosynthesis by maintaining glutamine/glutamate homeostasis [55]. Specifically, *gltB* (Die3_GM000254), *gltD* (Die3_GM000255), and *glnA* (Die3_GM001158) were upregulated by 1.3-, 1.7-, and 2.7-fold, respectively, when cells were under the C_16_ cultivation condition. Additionally, fatty acid CoA ligase FadD32 and polyketide synthase 13 are key enzymes involved in the biosynthesis of mycolic acids (MAs). MAs in the cell wall confer cell surface hydrophobicity, facilitating bacterial hydrocarbon adsorption [56]. Based on the gene expression of *fadD32* (Die3_GM000310) and *pks13* (Die3_GM000311), there were no significant transcriptional differences in cells under the C_16_ and pristane cultivation conditions when compared with the glucose control. Together, most genes associated with the biosynthesis of surface proteins, PLG layer, and lipids were simultaneously upregulated in C_16_ but downregulated in pristane, hypothesizing substrate specificity and a correlation with their CSH values.

### 3.9. Transcription Regulation

As master orchestrators of gene expression, transcriptional regulators are critically involved in mediating bacterial acclimatization to environmental stressors and alkane metabolism. Using the transcriptomics approach, a number of alkane-induced transcriptional regulators have been revealed (Supplementary Materials, Table S8). AraC family transcriptional regulators, which modulate diverse cellular processes including carbon metabolism, pathogenesis, and stress responses, are among the most common transcriptional regulators. Members of the AraC family primarily function as transcriptional activators and only infrequently assume repressor roles [21,22,57]. In *D.* sp. DQ12-45-1b, the AraC family regulator CypR located upstream of the CYP153 gene cluster was found to activate the CYP153 promoter when using medium-chain *n*-alkanes (C_8_-C_14_). In this study, four AraC family transcriptional regulator genes were downregulated in C_16_ compared to glucose but upregulated in pristane, which implies that they might have distinct roles or mechanisms in *n*-alkane and branched alkane utilization.

TetR family proteins are widely distributed in bacteria, with certain members regulating amino acid metabolism, alkane and fatty acid metabolism, antibiotic production, and osmotic stress [21,22,58]. Based on the GenBank database, putative TetR family regulator genes were positioned downstream of *alkB* in several Gram-positive bacteria (e.g., *Mycobacterium*, *Nocardioides*, *Gordonia,* and *Rhodococcus*), suggesting a potential functional association between TetR and AlkB in alkane degradation among these organisms. In *D.* sp. DQ12-45-1b, the TetR family regulator AlkX binds cooperatively to the *alkW1* promoter, leading to transcriptional repression of *alkW1* in the presence of long-chain *n*-alkanes (C_10_-C_24_) [22]. Seventeen TetR family regulators were identified in this study, with largely inverse expression profiles between C_16_ and pristane treatments. Notably, a transcriptional regulator of the TetR family (Die3_GM000016) is located next to *alkB* (Die3_GM000017), suggesting that the transcriptional regulator may modulate the expression of the adjacent monooxygenase gene, which needs further experimental verification.

LuxR family transcriptional regulators are related to aromatic compounds’ metabolism, quorum sensing, and biosurfactant synthesis [59,60,61]. In Gram-positive bacteria of *Rhodococcus* sp. P14, the LuxR family regulator NarL located upstream of a CYP153 gene (*cyp108j1*), was found to repress the promotion of *cyp108j1* in polycyclic aromatic hydrocarbon degradation [60]. In Gram-negative bacteria of *P. fluorescens* SS101, LuxR-type regulator genes *massAR* and *massBCR* most likely repressed massetolide biosynthesis genes through genetic, chemical, and phenotypic analyses [61]. In the CN-3 strain, four LuxR family genes exhibited differential expression under C_16_ or pristane cultivation. Specifically, a LuxR family transcriptional regulator gene (Die3_GM000698) and a cob(I)alamin adenosyltransferase gene (Die3_GM000699) are upregulated in C_16_ versus glucose treatment. Their close genomic proximity to the enoyl-CoA hydratase *fadH* gene (Die3_GM000696) suggests a role in regulating fatty acid oxidation.

ArsR family transcriptional regulators, known to function as metal-sensing proteins governing metal ion metabolism and oxidative stress response in many bacterial species [62], exhibited differential expression under different carbon sources. Six ArsR-encoding genes were downregulated and one was upregulated in C_16_ compared to glucose, while this trend was reversed in pristane. The LysR family represents one of the most conserved and abundant transcriptional regulator families in bacteria, playing crucial roles in metabolic regulation, virulence control, oxidative stress response, and antibiotic resistance [63]. In strain CN-3, five LysR-type regulators were identified, including Die3_GM001964 (co-induced by C_16_ and pristane) and Die3_GM001419 (co-repressed by C_16_ and pristane). Eight MarR family genes exhibited differential expression under C_16_ or pristane. The MarR family mainly participates in oxidative stress response, antibiotic resistance, aromatic compound catabolism, and energy metabolism (TCA cycle, electron transport chain, etc.) [64]. Eight GntR family genes were found in strain CN-3 with differential expression under C_16_ or pristane. These proteins regulate the metabolism of carbon, nitrogen, and fatty acids and help combat nutritional stress or toxic substances [65]. In addition, the genes coding for protein families such as WhiB, IciR, CspA, XRE, MerR, and PadR [66,67,68] were induced by C_16_ and/or pristane. The above analysis demonstrates that strain CN-3 has evolved a complex regulatory system to optimize alkane uptake, catabolism, and cellular adaptation. Most transcriptional regulators involved in hydrocarbon metabolism and adaptation to hydrocarbon-derived environments exhibited responsiveness to alkane supplementation. Validating the potential functions and interactions of regulatory factors through chromatin immunoprecipitation sequencing (ChIP-seq), electrophoretic mobility shift assays (EMSA), and related technologies will be a key focus of our subsequent research.

**Figure 6:**
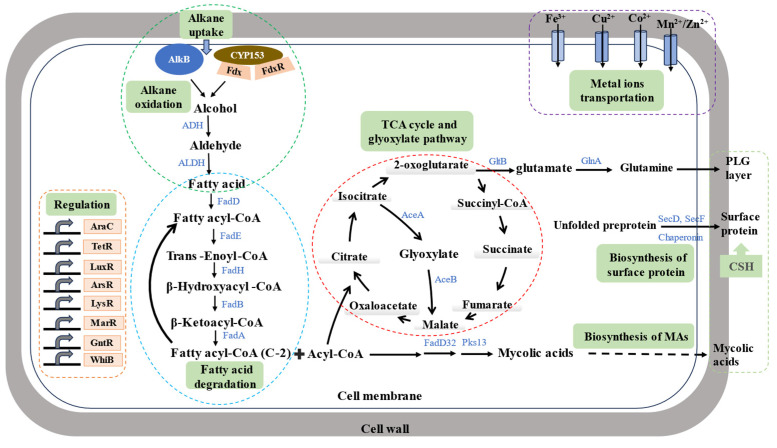
Scheme of comparative transcriptional profiles of CN-3 under C_16_ and pristane growth conditions. The graph shows that C_16_ and pristane induce different sets of genes involved in alkane uptake, alkane oxidation, fatty acid degradation, the TCA cycle and glyoxylate pathway, metal ion transportation, cell surface composition biosynthesis, and regulation.

In this study, we revealed the scheme of comparative transcriptional profiles of CN-3 under C_16_ and pristane growth conditions (Figure 6). Despite the insights provided, this study has several limitations. Firstly, our transcriptomic data were obtained from batch cultures at a single time point, which may not capture the full dynamic regulation of the genes of interest. Secondly, future work employing continuous culturing or multi-omics (e.g., post-transcriptional, translational, and post-translational) integration could more comprehensively elucidate the regulatory networks [9,41,69]. Thirdly, although this study focuses on core alkane degradation and lipid metabolism, other critical processes—such as stress response mechanisms, and biofilm formation—remain underexplored. Further investigation into these areas could yield valuable insights into microbial adaptive strategies and enhance our understanding of alkane degradation [18,19,62,63]. Lastly, the physiological measurements were conducted under laboratory conditions, and their extrapolation to natural environments such as mixed hydrocarbon pollutants, variable salinity, or microbial community interactions [1,30,70], requires further investigation to enhance practical bioremediation potential.

## 4. Conclusions

A comparative transcriptome analysis revealed different adaptation mechanisms of a salt-tolerant bacterium, *Dietzia* sp. CN-3, to utilize *n*-hexadecane (C_16_), branched alkane (pristane), and glucose. Both C_16_ and pristane induced common genes of alkane hydroxylation in the core alkane degradation pathway but elicited distinct transcriptional patterns of genes involved in lipid metabolism (fatty acid degradation), energy metabolism (TCA cycle and glyoxylate pathway), metal ion transportation, cell surface composition biosynthesis (surface proteins, PLG layer, and lipids), and transcription regulation. Comparative transcriptional analyses highlight that the CN-3 strain exhibited considerable metabolic plasticity to degrade alkanes with different chain lengths. This study provides critical insights into microbial alkane degradation, offering valuable information on petroleum bioremediation application.

## Figures and Tables

**Table 1:** Transcriptional profiles of genes associated with core alkane degradation pathway.

Functional	Gene ID	Function	C_16_/Glu	Pri/Glu	Pri/C_16_
Alkane uptake	Die3_GM000328	Glycosyltransferase	0.53	−0.16	−0.75
	Die3_GM000521	Glycosyltransferase family 2	0.57	−0.46	−1.08
	Die3_GM000522	Glycosyltransferase family 2	0.66	−1.18	−1.90
	Die3_GM002677	Glycosyltransferase	1.14	−0.45	−1.64
	Die3_GM003163	Glycosyltransferase	0.53	0.16	−0.43
	Die3_GM003164	Glycosyltransferase	−0.29	0.42	0.64
	Die3_GM000935	Lipoprotein	0.97	−1.76	−2.80
	Die3_GM003229	ABC transporter permease	−0.65	0.42	0.99
	Die3_GM000578	Adhesin	0.94	0.32	−0.69
Alkane hydroxylation	Die3_GM000017	Alkane hydroxylase-rubredoxin fusion protein, AlkB	4.09	−0.02	−4.17
	Die3_GM000105	Cytochrome P450, CYP153	4.75	1.15	−3.64
	Die3_GM000104	Ferredoxin	4.26	1.22	−3.07
	Die3_GM000106	Ferredoxin reductase	4.53	1.75	−3.64
	Die3_GM001585	Baeyer–Villiger monooxygenase	−0.48	0.24	0.65
	Die3_GM003194	Baeyer–Villiger monooxygenase	0.75	0.19	−0.63
Dehydrogenation of alcohols	Die3_GM000170	Alcohol dehydrogenase	1.98	−0.63	−2.67
	Die3_GM001258	Alcohol dehydrogenase	0.90	−0.48	−1.45
	Die3_GM001307	Alcohol dehydrogenase	1.40	−0.34	−1.80
	Die3_GM002689	Alcohol dehydrogenase	1.32	−0.36	−1.75
	Die3_GM002991	Alcohol dehydrogenase	0.51	−0.24	−0.81
	Die3_GM001956	Alcohol dehydrogenase	−0.27	0.25	0.45
	Die3_GM002554	Alcohol dehydrogenase	−0.97	0.08	0.99
Dehydrogenation of aldehydes	Die3_GM000037	Aldehyde dehydrogenase	0.58	−0.02	−0.67
	Die3_GM000266	Aldehyde dehydrogenase	0.61	−0.34	−1.01
	Die3_GM000241	Aldehyde dehydrogenase	−5.67	−4.11	1.51
	Die3_GM002359	Aldehyde dehydrogenase	−0.77	0.26	0.96
	Die3_GM002915	Aldehyde dehydrogenase	−0.39	0.02	0.35
	Die3_GM000896	Aldehyde dehydrogenase	−0.44	0.28	0.65
	Die3_GM002352	Aldehyde dehydrogenase	−0.82	0.38	1.14

The numbers in “C_16_/Glu”, “Pri/Glu”, and “Pri/C_16_” represent the log_2_FC values of “C_16_ versus glucose”, “pristane versus glucose”, and “pristane versus C_16_”. “−” indicates that the gene is downregulated.

**Table 2:** Transcriptional profiles of genes related to TCA cycle and glyoxylate pathway.

Pathways	Gene ID	Functional Annotation	C_16_/Glu	Pri/Glu	Pri/C_16_
TCA cycle	Die3_GM000733	Citrate synthase	0.77	−1.60	−2.41
	Die3_GM001512	Aconitase	0.94	−0.77	−1.78
	Die3_GM001914	Isocitrate dehydrogenase	1.10	−0.66	−1.82
	Die3_GM002241	Succinyl-CoA synthetase, alpha subunit	0.38	−1.60	−2.05
	Die3_GM002242	Succinyl-CoA synthetase, beta subunit	0.25	−1.79	−2.09
	Die3_GM000809	Succinate dehydrogenase iron–sulfur subunit	−0.29	−2.16	−1.92
	Die3_GM000810	Succinate dehydrogenase flavoprotein subunit	−0.06	−1.16	−1.16
	Die3_GM000811	Succinate dehydrogenase membrane anchor subunit	0.06	−1.22	−1.33
	Die3_GM000812	Succinate dehydrogenase cytochrome b subunit	0.17	−1.54	−1.76
	Die3_GM001943	Fumarase	1.35	−1.00	−2.41
	Die3_GM001000	Malate dehydrogenases	0.94	−0.42	−1.42
Glyoxylate pathway	Die3_GM002700	Isocitrate lyase (AceA)	0.66	−2.23	−2.94
	Die3_GM001647	Malata synthetase (AceB)	1.36	−0.84	−2.26

The numbers in “C_16_/Glu”, “Pri/Glu”, and “Pri/C_16_” represent the log_2_FC values of “C_16_ versus glucose”, “pristane versus glucose”, and “pristane versus C_16_”. “−” indicates that the gene is downregulated.

## Data Availability

The original contributions presented in this study are included in the article and Supplementary Materials. Further inquiries can be directed to the corresponding author.

## Supplementary Materials

The following supporting information can be downloaded at: https://www.mdpi.com/article/10.3390/microorganisms13092206/s1, Figure S1: The growth curves of strain CN-3 under glucose (A), C_16_ (A), and pristane (B) growth conditions; Figure S2: The correlation between samples under pristane (Pri), glucose (Glu), and C_16_ in the CN-3 strain; Figure S3: RT-qPCR validation of the RNA-Seq data; Figure S4: Comparative Venn analysis revealing the up- and downregulated DEGs in the C_16_ and pristane (Pri) groups when compared with the glucose control; Figure S5: Phylogenetic analysis of alcohol dehydrogenases (ADHs) from hydrocarbon-degrading bacteria; Figure S6: Multiple sequence alignment of predicted amino acid sequences with aldehyde dehydrogenases (ALDHs) from alkane-degrading bacteria using Bioedit software; Table S1: Primers used for RT-qPCR; Table S2: Primers used in the construction of Δ*alkB* and Δ*CYP153* mutants; Table S3: Data obtained from RNA-Seq; Table S4: Transcriptional profiles of genes related to fatty acid metabolism; Table S5: Transcriptional profiles of genes related to biomass synthesis; Table S6: Transcriptional profiles of genes related to metal ion transportation; Table S7: Transcriptional profiles of genes related to cell surface composition biosynthesis; Table S8: List of transcriptional regulators.

## References

[1] Vauloup A., Cébron A. (2025). Development of a device to trap soil bacteria capable of degrading organic contaminants such as alkanes and polycyclic aromatic hydrocarbons. J. Hazard. Mater..

[2] Rojo F. (2009). Degradation of alkanes by bacteria. Environ. Microbiol..

[3] Lea-Smith D.J., Biller S.J., Davey M.P., Cotton C.A., Perez Sepulveda B.M., Turchyn A.V., Scanlan D.J., Smith A.G., Chisholm S.W., Howe C.J. (2015). Contribution of cyanobacterial alkane production to the ocean hydrocarbon cycle. Proc. Natl. Acad. Sci. USA.

[4] Li Y., Liu Y., Guo D.Y., Dong H.L. (2024). Differential degradation of petroleum hydrocarbons by *Shewanella putrefaciens* under aerobic and anaerobic conditions. Front. Microbiol..

[5] Mu B.Z., Nazina T.N. (2022). Recent advances in petroleum microbiology. Microorganisms.

[6] Gaid M., Pöpke D., Reinhard A., Berzhanova R., Mukasheva T., Urich T., Mikolasch A. (2023). Characterization of the mycoremediation of *n*-alkanes and branched-chain alkanes by filamentous fungi from oil-polluted soil samples in Kazakhstan. Microorganisms.

[7] Shou L.B., Liu Y.F., Zhou J., Liu Z.L., Zhou L., Liu J.F., Yang S.Z., Gu J.D., Mu B.Z. (2021). New evidence for a hydroxylation pathway for anaerobic alkane degradation supported by analyses of functional genes and signature metabolites in oil reservoirs. AMB Express.

[8] Dawson K.S., Schaperdoth I., Freeman K.H., Macalady J.L. (2013). Anaerobic biodegradation of the isoprenoid biomarkers pristane and phytane. Org. Geochem..

[9] Xu H.X., Tang Y.Q., Nie Y., Wu X.L. (2022). Comparative transcriptome analysis reveals different adaptation mechanisms for degradation of very long-chain and normal long-chain alkanes in *Dietzia* sp. DQ12-45-1b. Environ. Microbiol..

[10] Feng L., Wang W., Cheng J.S., Ren Y., Zhao G., Gao C.X., Tang Y., Liu X.Q., Han W.Q., Peng X. (2007). Genome and proteome of long-chain alkane degrading *Geobacillus thermodenitrificans* NG80-2 isolated from a deep-subsurface oil reservoir. Proc. Natl. Acad. Sci. USA.

[11] Throne-Holst M., Wentzel A., Ellingsen T.E., Kotlar H.K., Zotchev S.B. (2007). Identification of novel genes involved in long-chain *n*-alkane degradation by *Acinetobacter* sp. strain DSM 17874. Appl. Environ. Microbiol..

[12] Wang W.P., Shao Z.Z. (2012). Genes involved in alkane degradation in the *Alcanivorax hongdengensis* strain A-11-3. Appl. Microbiol. Biotechnol..

[13] Wang W.P., Shao Z.Z. (2012). Diversity of flavin-binding monooxygenase genes (*almA*) in marine bacteria capable of degradation long-chain alkanes. FEMS Microbiol. Ecol..

[14] Wang W.P., Shao Z.Z. (2013). Enzymes and genes involved in aerobic alkane degradation. Front. Microbiol..

[15] Liu C.L., Wang W.P., Wu Y.H., Zhou Z.W., Lai Q.L., Shao Z.Z. (2011). Multiple alkane hydroxylase systems in a marine alkane degrader, *Alcanivorax dieselolei* B-5. Environ. Microbiol..

[16] Nie Y., Liang J.L., Fang H., Tang Y.Q., Wu X.L. (2014). Characterization of a CYP153 alkane hydroxylase gene in a gram-positive *Dietzia* sp. DQ12-45-1b and its “team role” with *alkW1* in alkane degradation. Appl. Microbiol. Biotechnol..

[17] Wang X.B., Nie Y., Tang Y.Q., Wu G., Wu X.L. (2013). Alkane chain length alters *Dietzia* sp. strain DQ12-45-1b biosurfactant production and cell surface activity. Appl. Environ. Microbiol..

[18] Zhang H., Zhang W.C., Zong Y.W., Kong D.Y., Ma L.Y., Wu X.L., Zhao K. (2024). Dynamics of microbial-induced oil degradation at the microscale. Microbiol. Spectr..

[19] Elumalai P., Parthipan P., AlSalhi M.S., Huang M., Devanesan S., Karthikeyan O.P., Kim W., Rajasekar A. (2021). Characterization of crude oil degrading bacterial communities and their impact on biofilm formation. Environ. Pollut..

[20] Wang W., Shao Z. (2014). The long-chain alkane metabolism network of *Alcanivorax dieselolei*. Nat. Commun..

[21] Liang J.L., JiangYang J.H., Nie Y., Wu X.L., Löffler F.E. (2016). Regulation of the alkane hydroxylase CYP153 gene in a gram-positive alkane-degrading bacterium, *Dietzia* sp. strain DQ12-45-1b. Appl. Environ. Microbiol..

[22] Liang J.L., Nie Y., Wang M., Xiong G., Wang Y.P., Maser E., Wu X.L. (2016). Regulation of alkane degradation pathway by a TetR family repressor via an autoregulation positive feedback mechanism in a gram-positive *Dietzia* bacterium. Mol. Microbiol..

[23] Ji N.N., Wang X.L., Yin C., Peng W.L., Liang R.B. (2019). CrgA protein represses AlkB2 monooxygenase and regulates the degradation of medium-to-long-chain *n*-alkanes in *Pseudomonas aeruginosa* SJTD-1. Front. Microbiol..

[24] Gunasekera T.S., Striebich R.C., Mueller S.S., Strobel E.M., Ruiz O.N. (2013). Transcriptional profiling suggests that multiple metabolic adaptations are required for effective proliferation of *Pseudomonas aeruginosa* in jet fuel. Environ. Sci. Technol..

[25] Li S.W., Huang Y.X., Liu M.Y. (2020). Transcriptome profiling reveals the molecular processes for survival of *Lysinibacillus fusiformis* strain 15-4 in petroleum environments. Ecotoxicol. Environ. Saf..

[26] Rainey F.A., Klatte S., Kroppenstedt R.M., Stackebrandt E. (1995). *Dietzia*, a new genus including *Dietzia maris* comb. nov., formerly *Rhodococcus maris*. Int. J. Syst. Bacteriol..

[27] Sun J.Q., Xu L., Zhang Z., Li Y., Tang Y.Q., Wu X.L. (2014). Diverse bacteria isolated from microtherm oil-production water. Antonie Van Leeuwenhoek.

[28] Hu B., Wang M., Geng S., Wen L., Wu M., Nie Y., Tang Y.Q., Wu X.L. (2020). Metabolic exchange with non-alkane-consuming *Pseudomonas stutzeri* SLG510A3-8 improves *n*-alkane biodegradation by the alkane degrader *Dietzia* sp. strain DQ12-45-1b. Appl. Environ. Microbiol..

[29] Gurav R., Lyu H., Ma J., Tang J., Liu Q., Zhang H. (2017). Degradation of *n*-alkanes and PAHs from the heavy crude oil using salt-tolerant bacterial consortia and analysis of their catabolic genes. Environ. Sci. Pollut. Res. Int..

[30] Chen W.W., Sun J.W., Ji R.P., Min J., Wang L.Y., Zhang J.W., Qiao H.J., Cheng S.W. (2024). Crude oil biodegradation by a biosurfactant-producing bacterial consortium in high-salinity soil. J. Mar. Sci. Eng..

[31] Chen W.W., Li J.D., Sun X.N., Min J., Hu X.K. (2017). High efficiency degradation of alkanes and crude oil by a salt-tolerant bacterium *Dietzia* species CN-3. Int. Biodeterior. Biodegrad..

[32] Wang W., Cai B., Shao Z.Z. (2014). Oil degradation and biosurfactant production by the deep sea bacterium *Dietzia maris* as-13-3. Front. Microbiol..

[33] Rosenberg M. (1984). Bacterial adherence to hydrocarbons: A useful technique for studying cell surface hydrophobicity. FEMS Microbiol. Lett..

[34] Trapnell C., Hendrickson D.G., Sauvageau M., Goff L., Rinn J.L., Pachter L. (2013). Differential analysis of gene regulation at transcript resolution with RNA-seq. Nat. Biotechnol..

[35] Livak K.J., Schmittgen T.D. (2001). Analysis of relative gene expression data using real-time quantitative PCR and the 2^−ΔΔC^_T_ method. Methods.

[36] Gunasekera T.S., Bowen L.L., Zhou C.E., Howard-Byerly S.C., Foley W.S., Striebich R.C., Dugan L.C., Ruiz O.N. (2017). Transcriptomic analyses elucidate adaptive differences of closely related strains of *Pseudomonas aeruginosa* in fuel. Appl. Environ. Microbiol..

[37] Mounier J., Hakil F., Branchu P., Naitali M., Goulas P., Sivadon P., Grimaud R. (2018). AupA and AupB are outer and inner membrane proteins involved in alkane uptake in *Marinobacter hydrocarbonoclasticus* SP17. MBio.

[38] Sabirova J.S., Becker A., Lünsdorf H., Nicaud J.-M., Timmis K.N., Golyshin P.N. (2011). Transcriptional profiling of the marine oil-degrading bacterium *Alcanivorax borkumensis* during growth on *n*-alkanes. FEMS Microbiol. Lett..

[39] Saurin W., Dassa E. (1994). Sequence relationships between integral inner membrane proteins of binding protein-dependent transport systems: Evolution by recurrent gene duplications. Protein Sci..

[40] Gregson B.H., Metodieva G., Metodiev M.V., Golyshin P.N., McKew B.A. (2018). Differential protein expression during growth on medium versus long-chain alkanes in the obligate marine hydrocarbon-degrading bacterium *Thalassolituus oleivorans* MIL-1. Front. Microbiol..

[41] Gregson B.H., Metodieva G., Metodiev M.V., McKew B.A. (2019). Differential protein expression during growth on linear versus branched alkanes in the obligate marine hydrocarbon-degrading bacterium *Alcanivorax borkumensis* SK2^T^. Environ. Microbiol..

[42] Li L.X., Zhang Y.Y., Huang X.F., He M.F., Liu J., Lu L.J., Cai C., Peng K.M. (2022). Transcriptomic insights into lower biomass and higher cell-surface hydrophobicity of *Dietzia natronolimnaea* S-XJ-1 grown on alkanes compared to fatty acid esters. Int. Biodeterior. Biodegrad..

[43] Park C., Shin B., Jung J., Lee Y., Park W. (2017). Metabolic and stress responses of *Acinetobacter oleivorans* DR1 during long-chain alkane degradation. J. Microbial Biotechnol..

[44] Fang H., Xu J.B., Nie Y., Wu X.L. (2021). Pangenomic analysis reveals that the evolution of *Dietzia* species depends on their living habitats. Environ. Microbiol..

[45] Sato M., Torres-Bacete J., Sinha P.K., Matsuno-Yagi A., Yagi T. (2014). Essential regions in the membrane domain of bacterial complex I (NDH-1): The machinery for proton translocation. J. Bioenerg. Biomembr..

[46] Cook G.M., Hards K., Vilcheze C., Hartman T., Berney M. (2014). Energetics of respiration and oxidative phosphorylation in mycobacteria. Microbiol. Spectr..

[47] O’Donnell A., Harvey L.M., McNeil B. (2011). The roles of the alternative NADH dehydrogenases during oxidative stress in cultures of the filamentous fungus aspergillus Niger. Fungal Biol..

[48] Ahn S., Jung J., Jang I.A., Madsen E.L., Park W. (2016). Role of glyoxylate shunt in oxidative stress response. J. Biol. Chem..

[49] Park C., Shin B., Park W. (2019). Alternative fate of glyoxylate during acetate and hexadecane metabolism in *Acinetobacter oleivorans* DR1. Sci. Rep..

[50] Sritharan M. (2016). Iron homeostasis in mycobacterium tuberculosis: Mechanistic insights into sideropho-remediated iron uptake. J. Bacteriol..

[51] Parus A., Ciesielski T., Woźniak-Karczewska M., Ślachciński M., Owsianiak M., Ławniczak Ł., Loibner A.P., Heipieper H.J., ’Chrzanowski Ł. (2023). Basic principles for biosurfactant-assisted (bio)remediation of soils contaminated by heavy metals and petroleum hydrocarbons-A critical evaluation of the performance of rhamnolipids. J. Hazard. Mater..

[52] Huang X., Peng K., Lu L., Wang R., Liu J. (2014). Carbon source dependence of cell surface composition and demulsifying capability of *Alcaligenes* sp. S-XJ-1. Environ. Sci. Technol..

[53] Fagan R.P., Fairweather N.F. (2014). Biogenesis and functions of bacterial S-layers. Nat. Rev. Microbiol..

[54] Tsukazaki T. (2018). Structure-based working model of SecDF, a proton-driven bacterial protein translocation factor. FEMS Microbiol. Lett..

[55] Aldarini N., Alhasawi A.A., Thomas S.C., Appanna V.D. (2017). The role of glutamine synthetase in energy production and glutamine metabolism during oxidative stress. Antonie Leeuwenhoek.

[56] Marrakchi H., Lanéelle M., Daffé M. (2014). Mycolic Acids: Structures, biosynthesis, and beyond. Chem. Biol..

[57] Gallegos M.T., Schleif R., Bairoch A., Hofmann K., Ramos J.L. (1997). AraC/XylS family of transcriptional regulators. Microbiol. Mol. Biol. Rev..

[58] Xu Z., Wang M., Ye B.C. (2017). TetR family transcriptional regulator pccd negatively controls propionyl coenzyme a assimilation in *Saccharopolyspora erythraea*. J. Bacteriol..

[59] Segura A., Molina L. (2023). LuxR402 of *Novosphingobium* sp. HR1a regulates the correct configuration of cell envelopes. Front. Microbiol..

[60] Kan J., Peng T., Huang T.W., Xiong G.M., Hu Z. (2020). NarL, a Novel repressor for CYP108j1 expression during PAHs degradation in *Rhodococcus* sp. P14. Int. J. Mol. Sci..

[61] Song C.X., Aundy K., van de Mortel J., Raaijmakers J.M. (2014). Discovery of new regulatory genes of lipopeptide biosynthesis in *Pseudomonas fluorescens*. FEMS Microbiol. Lett..

[62] Ren S., Li Q., Xie L., Xie J. (2017). Molecular mechanisms underlying the function diversity of ArsR family metalloregulator. Crit. Rev. Eukaryot. Gene Expression.

[63] Maddocks S.E., Oyston P.C. (2008). Structure and function of the LysR-type transcriptional regulator (LTTR) family proteins. Microbiology.

[64] Grove A. (2013). MarR family transcription factors. Curr. Biol..

[65] Mihasan M., Stefan M., Hritcu L., Artenie V., Brandsch R. (2013). Evidence of a plasmid-encoded oxidative xylose-catabolic pathway in *Arthrobacter nicotinovorans* pAO1. Res. Microbiol..

[66] Hong Y.H., Deng M.C., Xu X.M., Wu C.F., Xiao X., Zhu Q., Sun X.X., Zhou Q.Z., Peng J., Yuan J.P. (2016). Characterization of the transcriptome of *Achromobacter* sp. HZ01 with the outstanding hydrocarbon-degrading ability. Gene.

[67] Sharma V., Hardy A., Luthe T., Frunzke J. (2021). Phylogenetic distribution of WhiB- and Lsr2-Type regulators in *Actinobacteriophage* genomes. Microbiol. Spectr..

[68] Bez C., Javvadi S.G., Bertani I., Devescovi G., Guarnaccia C., Studholme D.J., Geller A.M., Levy A., Venturi V. (2020). AzeR, a transcriptional regulator that responds to azelaic acid in *Pseudomonas nitroreducens*. Microbiology.

[69] Wei G., Li S., Ye S., Wang Z., Zarringhalam K., He J., Wang W.P., Shao Z.Z. (2022). High-resolution small RNAs landscape provides insights into alkane adaptation in the marine alkane-degrader *Alcanivorax dieselolei* B-5. Int. J. Mol. Sci..

[70] Wang T.T., Jing J.W., Huang P.F., Guo X.Y., Li C., Qu Y.Y. (2025). Bioremediation of alkane-containing saline soils using the long-chain alkane-degrading bacterium *Pseudomonas aeruginosa* DL: Effects, communities, and networks. J. Hazard. Mater..

