# Supplementary material for: Comparative Transcriptomics Reveals Distinct Adaptation Mechanisms for Degradation of n-Alkane and Branched Alkane in the Salt-Tolerant Bacterium Dietzia sp. CN-3
Source: Microorganisms. 2025 Sep 20;13(9):2206. doi: 10.3390/microorganisms13092206 (PMC12472514; doi:10.3390/microorganisms13092206)
Supplement: Supplementary file 1 [file microorganisms-13-02206-s001.zip › microorganisms-3847902-supplementary.pdf]

microorganisms

MDPI

Article

Comparative Transcriptomics Reveals Distinct Adaptation Mechanisms for Degradation of *n*-Alkane and Branched Alkane in the Salt-Tolerant Bacterium *Dietzia* sp. CN-3

Weiwei Chen [1], Jiawei Sun [1], Xin Zhang [1], Jiawen Zhang [1], Yuan Wang [2] and Shiwei Cheng [1,*]

[1] School of Life Sciences, Ludong University, Yantai 264025, China; wwchen@ldu.edu.cn (W.C.); jwsun163@163.com (J.S.); 15192081793@163.com (X.Z.); jiawen66688@163.com (J.Z.)
[2] Yantai Institute of Coastal Zone Research, Chinese Academy of Sciences, Yantai 264003, China; yuanwang@yic.ac.cn
* Correspondence: swcheng@ldu.edu.cn

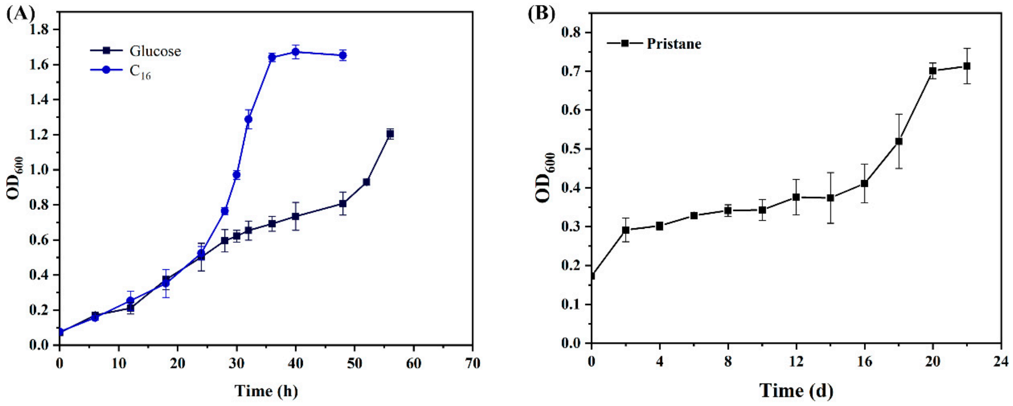

Figure S1. The growth curves of strain CN-3 under glucose (A), C_{16} (A), and pristane (B) growth conditions. Error bars indicate the standard deviation of three biological replicates.

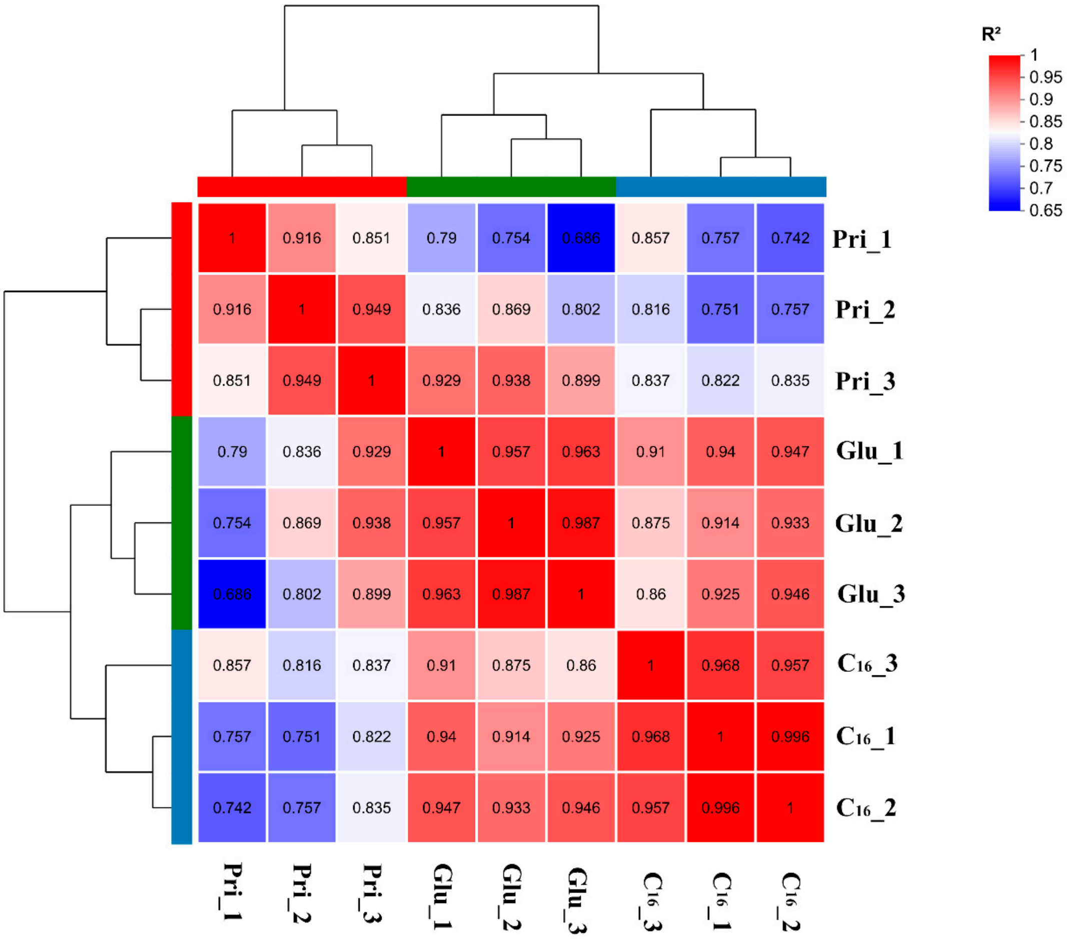

Figure S2. The correlation between samples under pristane (Pri), glucose (Glu), and C_{16} in the CN-3 strain. 1, 2 and 3 represent three biological replicates in each group.

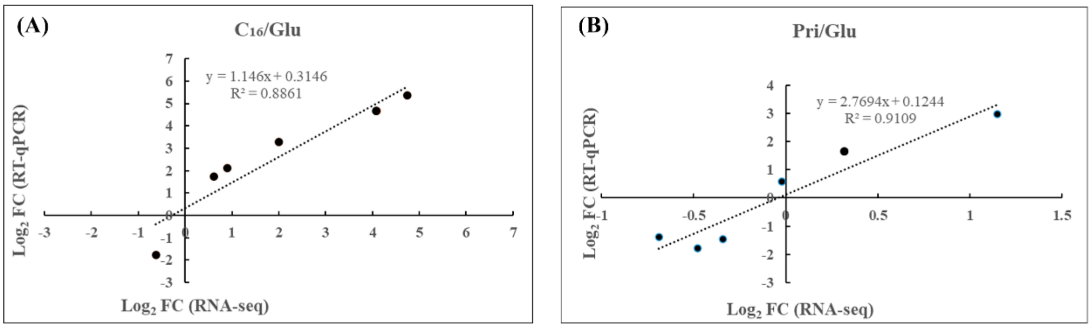

Figure S3. RT-qPCR validation of the RNA-Seq data. A strong correlation of coefficient (R^2) from C_{16} compared with glucose (C_{16}/Glu) and pristane compared with glucose (Pri/Glu) are observed. The 16S rRNA gene was selected as the reference.

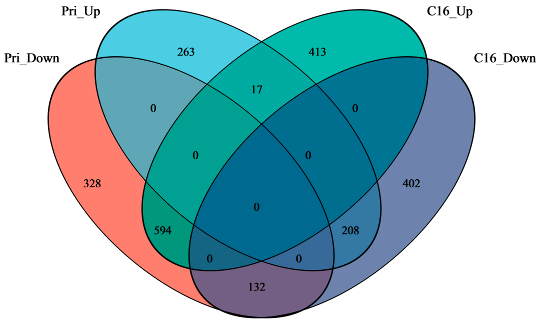

Figure S4. Comparative venn analysis revealing the up- and downregulated DEGs in the C16 and pristane (Pri) groups when compared with the glucose control.

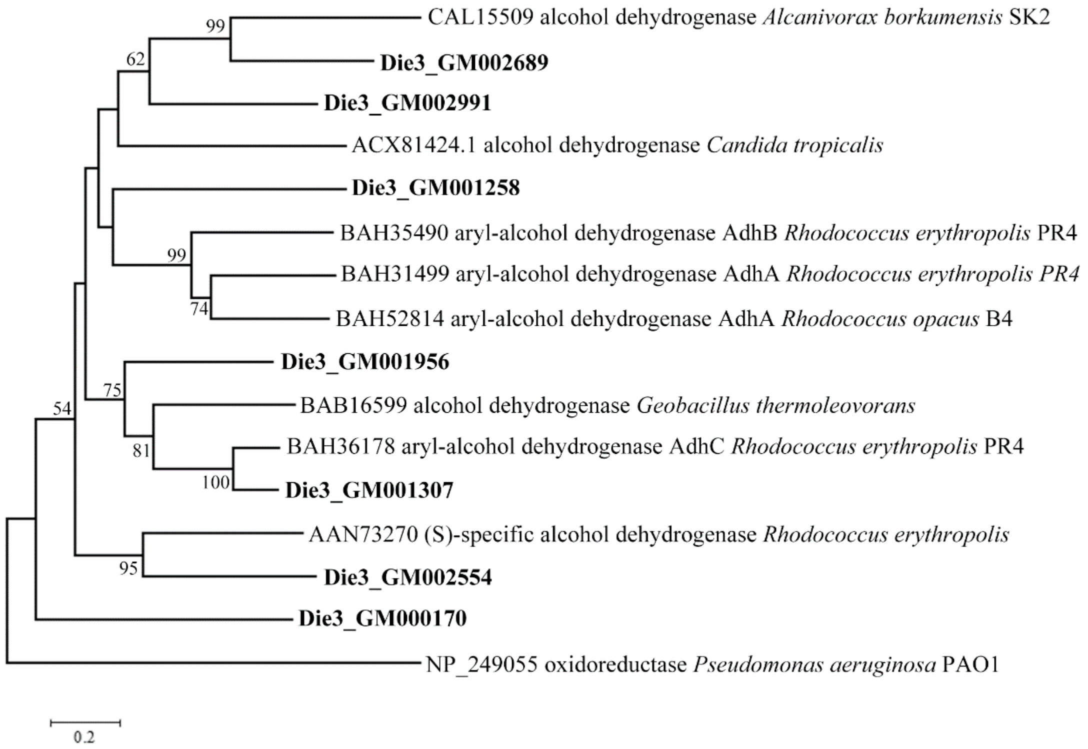

Figure S5. Phylogenetic analysis of alcohol dehydrogenases (ADHs) from hydrocarbon-degrading bacteria. The bootstrap values are shown at branch points (1000 replications). The access numbers used to construct the phylogenetic tree are based on the protein database in National Center for Biotechnology Information. Seven ADHs homologous genes are presented in bold letters. Bar indicates 0.20 substitutions per nucleotide position.

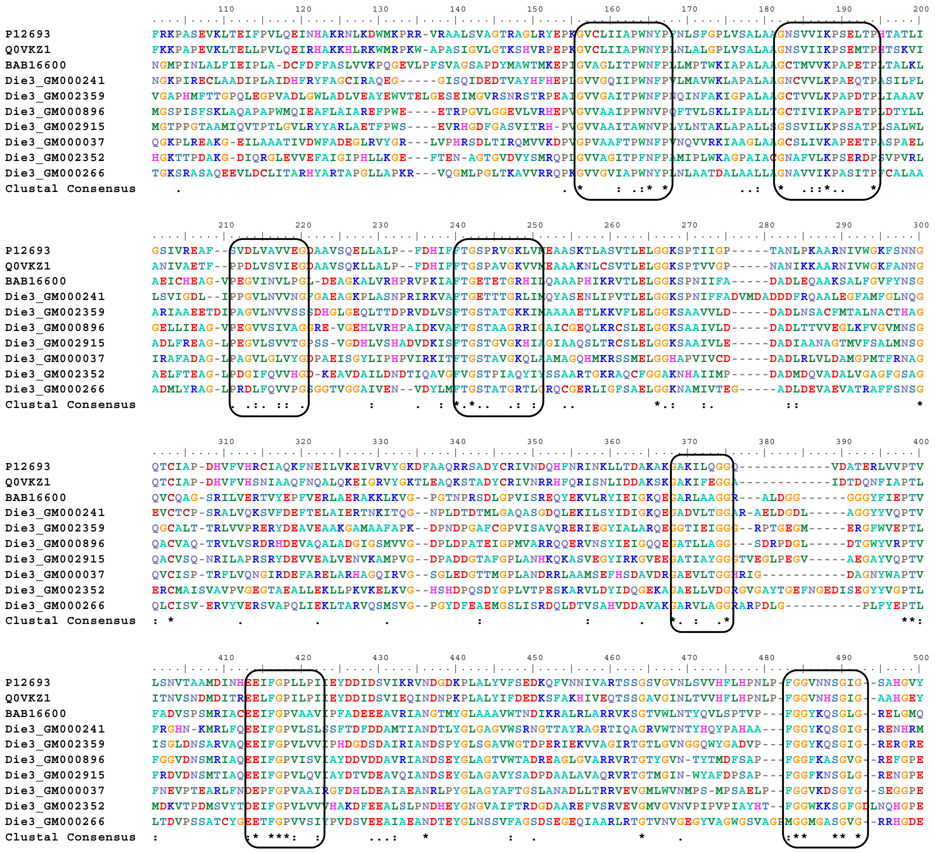

Figure S6. Multiple sequence alignment of predicted amino acid sequences with aldehyde dehydrogenases (ALDHs) from alkane-degrading bacteria using Bioedit software. Seven ALDH homologous genes including Die3_GM000241, Die3_GM002359, Die3_GM000896, Die3_GM002915, Die3_GM000037, Die3_GM002352 and Die3_GM000266 from CN-3, P12693 from *Pseudomonas oleovorans*, Q0VKZ1 from *Alcanivorax borkumensis* SK2, BAB16600 from *Geobacillus thermoleovorans* B23. Asterisks (*) and dots (.) represent identical and similar residues, respectively; double dots (:) indicate higher similarity. The boxed residues comprise conserved motifs for the ALDHs function

Table S1. Primers used for RT-qPCR.

Primer	Sequence (5′-3′)	Purpose
**RT-alkB-F**	GCCAACATCCAGCCGTCCAA	To amplify partial fragment of *alkB* for RT-qPCR
**RT-alkB-R**	CCGAGTAGTGGTTCCCGCAGTT	
**RT-CYP-F**	CAAGGACGAATGGGAAGAA	To amplify partial fragment of *CYP153* for RT-qPCR
**RT-CYP-R**	CTGTTGGGCTGAAAGTGGA	
**RT-fdx-F**	TCGGGAGCAAGTGAAGAAG	To amplify partial fragment of ferredoxin for RT-qPCR
**RT-fdx-R**	AACTCTGGCACCTGGACGGTAA	
**RT-FdR-F**	GCCAAAGTCGCAGCATTA	To amplify partial fragment of ferredoxin reductase for RT-qPCR
**RT-FdR-R**	ACCATCGTTCAGGGTCAAGC	
**RT-16S-F**	CGTCGTCTGTGAAATCCCTCG	To amplify partial fragment of 16S rRNA for RT-qPCR
**RT-16S-R**	TCAGCGTCAGTTACTACCCAGAG	

Table S2. Primers used in the construction of Δ*alkB* and Δ*CYP153* mutants.

Primer	Sequence (5′-3′)	Purpose
**Dis-A-F**	ATATCCCGGGACCTCTGGTCCGCCGACGA	To amplify homologous fragment of *alkB* for gene knockout
**Dis-A-R**	ACTGAAGCTTTGCTCGAAGCTGCGCAGTA	
**Ver-A-F**	AGCCTATGGAAAAACGCCAG	To amplify partial fragment for Δ*alkB* verification
**Ver-A-R**	GTAAAACGACGGCCAGTG	
**Dis-C-F**	ATATCCCGGGTCTTCTCCGCCGAGCCGAT	To amplify homologous fragment of *CYP153* for gene knockout
**Dis-C-R**	ACTGAAGCTTATGTAGGCCAGCGGCGTTTG	
**Ver-C-F**	CAGGAAACAGCTATGACCAT	To amplify partial fragment for Δ*CYP153* verification
**Ver-C-R**	GTAAAACGACGGCCAGTG	

*Specified restriction sites are underlined.

Table S3. Data obtained from RNA-Seq.

Sample	Total raw reads	Total clean reads	Genome Mapped Reads	Genome mapping ratio
Glu_1	40280294	39258372	28492482	72.58
Glu_2	28038674	27463172	22749856	82.84
Glu_3	24365760	23935040	23391402	97.73
C16_1	21348834	20876008	20306285	97.27
C16_2	24917138	24357880	23604448	96.91
C16_3	24955684	24381718	23613464	96.85
Pri_1	22040756	21547096	20835730	96.7
Pri_2	25083832	24505462	23247365	94.87
Pri_3	24931568	24442794	24026994	98.3

Table S4. Transcriptional profiles of genes related to fatty acid metabolism.

Gene	Gene ID	C_{16}/Glu	FDR	Pri/Glu	FDR	Pri/C_{16}	FDR
fadD	Die3_GM000231	2.02	2.01E-18	−1.24	5.24E-07	−3.33	1.87E-75
	Die3_GM000310	1.01	3.82E-07	−0.25	3.99E-02	−1.32	9.96E-30
	Die3_GM002675	0.42	3.80E-03	0.14	2.51E-02	−0.34	2.79E-04
	Die3_GM000171	−0.73	1.77E-04	0.13	3.04E-02	0.80	4.04E-11
	Die3_GM002355	−0.62	2.71E-03	0.32	1.48E-02	0.88	2.29E-28
	Die3_GM002836	−0.66	2.67E-04	0.20	4.65E-02	0.80	1.13E-24
	Die3_GM003272	−0.58	6.34E-04	0.08	4.61E-03	0.60	4.29E-17
fadE	Die3_GM000063	0.13	7.08E-03	0.36	3.28E-02	0.19	1.54E-02
	Die3_GM000839	1.20	1.08E-06	−1.43	2.86E-20	−2.70	7.31E-38
	Die3_GM001179	2.64	3.40E-40	−0.22	7.50E-03	−2.91	3.09E-59
	Die3_GM002953	1.04	5.37E-08	−0.29	6.89E-03	−1.39	2.95E-27
	Die3_GM000073	−0.30	3.27E-02	0.31	3.44E-02	0.56	3.91E-05
	Die3_GM000511	−0.18	5.65E-03	0.31	1.52E-02	0.42	1.43E-03
	Die3_GM000899	−0.13	5.68E-03	0.95	9.22E-12	1.02	6.74E-16
	Die3_GM001958	−0.53	6.86E-03	0.42	2.76E-04	0.89	2.08E-19
	Die3_GM002336	−0.16	4.66E-03	−0.37	5.41E-03	−0.26	1.04E-02
	Die3_GM002339	−0.66	2.20E-03	0.17	1.77E-02	0.76	4.02E-11
	Die3_GM003144	−0.48	6.28E-03	0.27	1.44E-02	0.68	3.20E-05
fadH	Die3_GM000532	1.01	1.80E-08	−0.36	8.75E-05	−1.43	3.74E-47
	Die3_GM000696	1.11	5.39E-08	−0.17	2.04E-02	−1.34	6.39E-17
	Die3_GM000705	1.12	1.45E-04	−0.56	1.56E-04	−1.75	1.20E-09
	Die3_GM000064	−0.57	4.02E-02	0.15	4.91E-02	0.66	3.48E-06
	Die3_GM002335	−0.49	4.52E-02	0.09	6.55E-03	0.52	1.81E-06
	Die3_GM003273	−0.54	3.65E-02	0.34	5.73E-03	0.82	4.24E-08
fadB	Die3_GM000706	1.11	1.54E-04	−0.50	1.60E-04	−1.78	1.22E-09
	Die3_GM000973	1.09	1.66E-09	0.21	1.28E-02	−0.93	1.00E-22
	Die3_GM002699	−0.52	6.31E-03	−0.29	1.58E-02	0.17	9.39E-03
fadA	Die3_GM000704	0.81	1.68E-06	−0.45	4.09E-03	−1.31	9.57E-25
	Die3_GM001800	1.28	1.50E-11	−0.96	8.93E-19	−2.29	1.30E-52
	Die3_GM001946	0.88	1.36E-04	0.28	1.92E-02	−0.66	5.01E-10
	Die3_GM001982	1.43	8.16E-07	−0.61	5.23E-07	−2.10	6.47E-13
	Die3_GM002845	0.52	6.95E-03	−0.85	8.10E-17	−1.42	1.26E-33
	Die3_GM000066	−0.53	1.27E-02	0.22	1.29E-03	0.69	9.71E-09
	Die3_GM003451	−0.77	8.32E-05	0.04	8.20E-03	0.76	8.09E-07

The numbers in "C_{16}/Glu", "Pri/Glu" and "Pri/C_{16}" represent $\log_2$FC of "C_{16} versus glucose", "pristane versus glucose" and "pristane versus C_{16}". "−" indicates that gene is downregulated. FadD, fatty acyl-CoA synthese; FadE, acyl-CoA dehydrogenase; FadH, enoyl-CoA hydratase; FadB, 3-hydroxybutyryl-CoA dehydrogenase; FadA, acetyl-CoA acyltransferase.

Table S5. Transcriptional profiles of genes related to biomass synthesis.

Pathways	Gene ID	Functional annotation	C_{16}/Glu	FDR	Pri/Glu	FDR	Pri/C16	FDR
Complex **I**	Die3_GM000380	NADH-quinone oxidoreductase subunit N	−0.72	4.70E-03	0.20	3.60E-02	0.85	1.03E-10
	Die3_GM000381	NADH-quinone oxidoreductase subunit M	−0.59	1.66E-02	0.25	2.21E-02	0.77	7.46E-14
	Die3_GM000382	NADH-quinone oxidoreductase subunit L	−0.62	4.37E-03	0.24	1.28E-02	0.80	5.75E-14
	Die3_GM000383	NADH-quinone oxidoreductase subunit K	−0.02	9.81E-03	0.59	2.43E-02	0.57	4.02E-02
	Die3_GM000384	NADH-quinone oxidoreductase subunit J	−0.68	1.33E-02	0.24	2.77E-02	0.84	4.87E-10
	Die3_GM000385	NADH-quinone oxidoreductase subunit I	−0.56	2.03E-02	0.10	5.22E-03	0.60	2.02E-06
	Die3_GM000386	NADH-quinone oxidoreductase subunit H	−0.53	6.31E-03	0.30	9.62E-03	0.78	2.45E-13
	Die3_GM000387	NADH-quinone oxidoreductase subunit G	−0.66	1.27E-02	0.38	4.30E-02	0.97	6.69E-09
	Die3_GM000388	NADH-quinone oxidoreductase subunit F	−0.68	1.61E-03	0.22	1.22E-02	0.84	8.38E-17
	Die3_GM000389	NADH-quinone oxidoreductase subunit E	−0.74	1.22E-03	0.38	3.57E-03	1.07	1.14E-16
	Die3_GM000390	NADH-quinone oxidoreductase subunit D	−0.75	1.68E-04	0.14	2.92E-02	0.82	9.30E-17
	Die3_GM000391	NADH-quinone oxidoreductase subunit C	−0.68	5.02E-03	0.28	7.78E-03	0.91	1.09E-11
	Die3_GM000392	NADH-quinone oxidoreductase subunit B	−0.68	9.02E-03	0.14	5.67E-03	0.76	2.79E-07
	Die3_GM000393	NADH-quinone oxidoreductase subunit A	−1.02	1.22E-04	−0.24	3.36E-03	0.73	2.71E-06
Purine biosynthesis	Die3_GM000651	Phosphoribosylamine-glycine ligase	0.45	2.58E-02	0.06	7.17E-03	−0.46	5.19E-12
	Die3_GM000659	adenylosuccinate lyase	0.45	4.17E-02	−0.22	1.75E-02	−0.73	1.90E-12
	Die3_GM000662	phosphoribosylaminoimidazole-succinocarboxamide synthase	1.23	1.83E-13	−0.26	4.81E-02	−1.56	7.36E-69
	Die3_GM000668	phosphoribosylformylglycinamidine synthase subunit PurS	0.85	8.46E-04	−1.55	3.92E-08	−2.45	2.52E-64
	Die3_GM000669	phosphoribosylformylglycinamidine synthase subunit PurQ / glutaminase	1.09	2.05E-07	−0.34	2.02E-02	−1.49	3.92E-19
	Die3_GM000672	phosphoribosylformylglycinamidine synthase subunit PurL	0.51	1.96E-02	−0.85	2.43E-11	−1.42	1.95E-26
	Die3_GM000677	amidophosphoribosyltransferase	0.57	1.19E-03	−0.40	8.22E-05	−1.03	3.21E-36
	Die3_GM000678	phosphoribosylformylglycinamidine cyclo-ligase	0.55	2.70E-03	−0.43	5.69E-05	−1.03	1.15E-37

Cytochrome d/b biosynthesis	Die3_GM000915	NAD⁺ synthase	−0.61	1.04E-02	0.29	1.06E-02	0.83	7.51E-16
	Die3_GM000916	large subunit ribosomal protein L36	0.01	9.86E-03	−2.97	3.50E-05	−3.02	2.04E-06
	Die3_GM002112	ATP-binding cassette, subfamily C, bacterial CydCD	−0.22	2.81E-03	−0.09	5.33E-03	0.08	5.24E-03
	Die3_GM002115	cytochrome bd ubiquinol oxidase subunit I	0.31	2.37E-02	−1.73	1.03E-74	−2.10	3.55E-22
	Die3_GM002116	cytochrome bd ubiquinol oxidase subunit II	−0.24	3.33E-02	−1.50	4.30E-58	−1.32	1.19E-12
De novo DNA synthesis	Die3_GM000917	glutaredoxin-like protein NrdH	0.39	1.69E-02	−1.98	3.12E-17	−2.42	2.09E-27
	Die3_GM000918	protein involved in ribonucleotide reduction	0.53	1.56E-02	−0.62	1.85E-02	−1.19	1.74E-30
	Die3_GM000919	ribonucleoside-diphosphate reductase alpha chain	0.61	5.27E-03	−0.71	1.33E-15	−1.37	4.26E-15
	Die3_GM000920	ribonucleoside-diphosphate reductase beta chain	0.27	1.61E-02	−0.81	7.94E-11	−1.14	6.73E-40
	Die3_GM001256	ADP-dependent NAD(P)H-hydrate dehydratase	0.02	9.53E-03	0.09	6.14E-03	0.01	9.22E-03
	Die3_GM001642	transcriptional repressor NrdR	0.51	3.48E-02	−0.41	4.68E-02	−0.97	7.12E-11
Cytochrome c biosynthesis	Die3_GM001174	cytochrome c oxidase subunit II	0.26	3.67E-02	−2.49	2.42E-43	−2.81	1.81E-29
	Die3_GM001183	ubiquinol-cytochrome c reductase cytochrome b subunit	0.42	1.11E-02	−1.99	1.24E-82	−2.47	2.21E-29
	Die3_GM001184	ubiquinol-cytochrome c reductase iron-sulfur subunit	0.39	1.05E-02	−1.64	3.96E-48	−2.09	1.34E-26
	Die3_GM001185	ubiquinol-cytochrome c reductase cytochrome c subunit	0.79	9.12E-03	−1.37	3.59E-22	−2.23	4.41E-13
	Die3_GM001186	cytochrome c oxidase subunit III	0.41	7.84E-03	−2.19	9.22E-33	−2.66	1.40E-37
	Die3_GM001535	heme a synthase	0.46	3.71E-02	−0.12	4.96E-02	−0.64	1.01E-11
	Die3_GM001538	heme o synthase	−0.09	6.77E-03	−0.03	7.54E-03	0.00	9.86E-03
	Die3_GM002640	cytochrome c biogenesis protein	0.13	5.77E-03	0.19	1.50E-02	−0.01	9.68E-03
	Die3_GM002649	cytochrome c-type biogenesis protein	−0.15	5.75E-03	0.09	6.53E-03	0.18	2.13E-02
Pyrimidine biosynthesis	Die3_GM001571	orotidine-5'-phosphate decarboxylase	−0.28	2.69E-02	0.20	1.89E-02	0.42	4.98E-03
	Die3_GM001572	carbamoyl-phosphate synthase large subunit	0.27	2.87E-02	−0.43	1.45E-05	−0.76	5.97E-05

	Die3_GM001573	carbamoyl-phosphate synthase small subunit	0.16	5.03E-03	−0.41	1.40E-03	−0.63	1.89E-05
	Die3_GM001575	dihydroorotase	−0.02	9.49E-03	−0.09	5.50E-03	−0.14	2.81E-03
	Die3_GM001576	aspartate carbamoyltransferase catalytic subunit	0.04	8.87E-03	−0.13	3.36E-02	−0.22	2.11E-03
	Die3_GM001577	pyrimidine operon attenuation protein / uracil phosphoribosyltransferase	0.78	2.82E-02	−2.85	4.64E-15	−3.67	9.11E-21
	Die3_GM002782	adenylosuccinate synthase	−0.06	7.93E-03	−0.74	2.45E-08	−0.73	1.08E-10
ATP synthesis	Die3_GM001806	F-type H+-transporting ATPase subunit epsilon	1.52	2.15E-11	−1.88	1.61E-08	−3.44	7.01E-46
	Die3_GM001807	F-type H+/Na+-transporting ATPase subunit beta	0.69	1.80E-02	−1.68	2.82E-69	−2.43	1.36E-18
	Die3_GM001808	F-type H+-transporting ATPase subunit gamma	0.35	2.10E-02	−1.79	4.40E-10	−2.19	5.52E-21
	Die3_GM001809	F-type H+/Na+-transporting ATPase subunit alpha	0.29	2.81E-02	−1.77	4.48E-62	−2.12	1.11E-23
	Die3_GM001810	F-type H+-transporting ATPase subunit delta	0.35	1.94E-02	−1.43	1.70E-31	−1.83	2.78E-18
	Die3_GM001811	F-type H+-transporting ATPase subunit b	0.32	2.57E-02	−2.25	3.41E-40	−2.63	8.05E-32
	Die3_GM001812	F-type H+-transporting ATPase subunit c	0.70	3.58E-03	−1.61	2.58E-10	−2.36	2.31E-53
	Die3_GM001813	F-type H+-transporting ATPase subunit a	0.49	4.24E-02	−1.51	2.01E-15	−2.06	7.67E-32

The numbers in "C_{16}/Glu", "Pri/Glu" and "Pri/C_{16}" represent the log_2FC values of "C_{16} versus glucose", "pristane versus glucose" and "pristane versus C_{16}". "−" indicates that gene is downregulated.

Table S6. Transcriptional profiles of genes related to metal ion transportation.

Gene ID	Functional annotation	C_{16}/Glu	FDR	Pri/Glu	FDR	Pri/C_{16}	FDR
Die3_GM000786	ABC-type Mn^{2+}/Zn^{2+} transport system, permease component	−0.51	2.69E-02	0.25	8.08E-03	0.70	6.86E-10
Die3_GM000787	ABC-type Mn^{2+}/Zn^{2+} transport system, ATPase component	−0.35	2.42E-02	0.51	8.27E-03	0.79	2.46E-07
Die3_GM000788	ABC-type Zn uptake system ZnuABC, Zn-binding component ZnuA	−0.62	1.16E-02	0.31	7.96E-04	0.87	1.86E-12
Die3_GM003113	Copper chaperone CopZ	0.06	8.99E-03	−0.60	5.21E-03	−0.72	2.82E-02
Die3_GM001396	Mn-dependent transcriptional regulator MntR, DtxR family	1.98	3.83E-22	0.40	5.56E-14	−3.34	9.72E-97
Die3_GM001122	Cobalamin biosynthesis protein CobD/CbiB	−0.29	3.69E-02	0.37	1.46E-02	0.59	1.35E-04
Die3_GM001278	Biotin transport system permease protein	−0.65	2.32E-02	0.27	1.86E-02	0.85	9.02E-08
Die3_GM001279	Biotin transport system ATP-binding protein	−0.44	5.65E-03	0.20	1.60E-02	0.59	9.57E-08
Die3_GM001841	Mg^{2+} and Co^{2+} transporter CorA	−0.18	4.31E-04	0.19	1.08E-02	0.31	1.60E-03
Die3_GM000196	Molybdopterin synthase catalytic subunit MoaE	−0.09	6.94E-04	0.32	7.40E-03	0.35	6.89E-05
Die3_GM000197	Molybdenum cofactor biosynthesis enzyme MoaC	0.59	4.39E-03	−0.08	6.39E-03	−0.73	2.34E-18
Die3_GM000205	Molybdopterin Mo-transferase	−0.36	1.29E-02	0.26	1.56E-02	0.55	5.00E-10
Die3_GM000208	Molybdopterin synthase sulfur carrier subunit MoaD	0.13	7.16E-04	−2.12	2.64E-11	−2.30	7.91E-33
Die3_GM001077	Cobyrinic acid a,c-diamide synthase, cobB-cbiA	1.36	4.15E-10	0.13	5.30E-03	−1.29	2.37E-29
Die3_GM001078	Cob(I)alamin adenosyltransferase, cobA	1.72	2.23E-17	−1.15	5.22E-25	−2.93	4.58E-66
Die3_GM001079	Magnesium chelatase subunit D	1.32	3.07E-17	−0.45	1.01E-07	−1.82	3.20E-13
Die3_GM001075	Cobalt-precorrin-6A reductase, cobK-cbiJ	−0.33	2.69E-02	0.76	5.37E-06	1.03	1.39E-13
Die3_GM001080	Cobalt-precorrin-6B C5, C15-methyltransferase, cobL-cbiET	0.25	2.56E-02	0.28	4.05E-03	−0.03	6.34E-04
Die3_GM001081	Cobalt-precorrin-4 C11-methyltransferase, cobM	−0.13	6.64E-03	0.17	4.22E-03	0.24	1.06E-02
Die3_GM001112	Cobalt-precorrin-8 methylmutase, cobH-cbiC	1.14	1.94E-07	−0.27	1.34E-02	−1.47	2.70E-31
Die3_GM000457	Fur family transcriptional regulator, furA	−1.91	9.54E-14	−2.60	4.08E-16	−0.74	2.63E-03
Die3_GM002064	Cation diffusion facilitator family transporter, czcD	−0.26	5.05E-04	0.45	2.98E-02	0.68	1.16E-13

The numbers in "C_{16}/Glu", "Pri/Glu" and "Pri/C_{16}" represent the log_2FC values of "C_{16} versus glucose", "pristane versus glucose" and "pristane versus C_{16}". "−" indicates that gene is downregulated.

Table S7. Transcriptional profiles of genes related to cell-surface composition biosynthesis.

Pathways	Gene ID	Functional annotation	C_{16}/Glu	FDR	Pri/Glu	FDR	Pri/C_{16}	FDR
Biosynthesis of surface protein	Die3_GM001600	preprotein translocase subunit SecF	0.70	3.12E-03	−0.32	2.01E-02	−1.07	8.20E-16
	Die3_GM001601	preprotein translocase subunit SecD	0.78	8.80E-06	−0.29	1.33E-02	−1.13	4.73E-32
	Die3_GM000756	chaperonin GroES	0.96	1.45E-03	−1.54	4.81E-09	−2.56	1.60E-13
	Die3_GM000757	chaperonin GroEL	0.65	3.11E-03	−0.49	7.20E-04	−1.20	2.77E-14
Biosynthesis of PLG layer	Die3_GM000254	glutamate synthase (NADPH) large chain GltB	0.38	5.84E-03	−1.06	3.23E-35	−1.50	4.28E-29
	Die3_GM000255	glutamate synthase (NADPH) small chain GltD	0.79	2.24E-04	−1.01	7.90E-16	−1.86	2.25E-31
	Die3_GM000039	glutamine synthetase GlnA	−0.47	1.53E-02	0.23	3.99E-02	0.64	2.63E-12
	Die3_GM001158	glutamine synthetase GlnA	1.44	2.02E-08	−0.41	3.10E-05	−1.91	9.99E-14
Biosynthesis of cell-surface lipids	Die3_GM000310	fatty acid CoA ligase FadD32	1.00	3.82E-07	−0.258	4.00E-02	−1.32	9.96E-30
	Die3_GM000311	polyketide synthase 13 Pks13	−0.09	7.21E-03	−0.67	1.17E-07	−0.64	3.58E-12

The numbers in "C_{16}/Glu", "Pri/Glu" and "Pri/C_{16}" represent the log_2FC values of "C_{16} versus glucose", "pristane versus glucose" and "pristane versus C_{16}". "−" indicates that gene is downregulate.

Table S8. List of transcriptional regulators.

Protein family	Gene ID	C_{16}/Glu	FDR	Pri/Glu	FDR	Pri/C_{16}	FDR
AraC	Die3_GM000200	−0.52	4.36E-02	0.44	1.30E-02	0.89	1.77E-13
	Die3_GM000420	−0.50	5.85E-03	0.19	4.15E-03	0.63	6.20E-10
	Die3_GM001584	−0.44	3.29E-02	0.29	1.64E-02	0.67	7.52E-14
	Die3_GM003195	−0.31	1.98E-02	0.32	2.88E-02	0.57	5.96E-08
TetR	Die3_GM000016	2.00	1.18E-32	−0.69	6.66E-07	−2.74	1.41E-123
	Die3_GM000070	0.08	8.21E-04	−0.87	3.48E-03	−0.99	4.27E-12
	Die3_GM000157	−0.25	3.98E-04	0.41	2.04E-03	0.60	8.84E-06
	Die3_GM000223	−0.41	7.24E-04	0.22	1.82E-02	0.56	1.86E-11
	Die3_GM000371	1.58	4.23E-15	−0.50	1.29E-03	−2.14	2.49E-115
	Die3_GM000444	0.32	3.33E-03	−0.75	3.33E-06	−1.12	3.46E-05
	Die3_GM000650	0.08	7.63E-03	−2.13	9.06E-54	−2.27	8.54E-76
	Die3_GM000807	0.56	3.07E-03	0.27	5.19E-03	-0.35	3.64E-04
	Die3_GM000877	−0.49	2.83E-02	0.38	2.49E-02	0.81	4.94E-27
	Die3_GM001513	−0.31	3.58E-03	0.21	3.94E-03	0.45	1.18E-02
	Die3_GM001836	1.43	2.24E-11	−1.38	1.06E-14	−2.87	8.48E-43
	Die3_GM002127	−0.74	3.03E-03	0.32	5.13E-03	0.99	3.05E-12
	Die3_GM002358	0.46	4.69E-02	0.25	1.87E-02	−0.27	8.36E-03
	Die3_GM002496	−0.58	1.08E-02	0.21	2.20E-02	0.72	7.61E-13
	Die3_GM002519	−0.50	7.64E-03	0.37	5.84E-03	0.80	2.12E-08
	Die3_GM002881	0.59	8.42E-03	−0.24	2.10E-02	−0.88	3.77E-09
	Die3_GM003152	−0.45	5.23E-03	0.25	1.37E-02	0.63	8.51E-11
LuxR	Die3_GM000449	1.11	1.78E-06	−0.78	1.45E-03	−1.93	6.99E-46
	Die3_GM000698	0.57	3.98E-03	−0.46	1.78E-04	−1.09	4.58E-30
	Die3_GM000986	−0.45	7.87E-03	0.36	6.69E-03	0.74	4.75E-08
	Die3_GM002349	−0.39	1.15E-02	0.10	6.26E-03	0.42	1.96E-05
ArsR	Die3_GM000434	−0.40	4.18E-02	0.35	1.44E-02	0.70	1.61E-14
	Die3_GM001062	0.48	1.04E-02	−0.19	4.94E-03	−0.72	7.78E-06
	Die3_GM001083	−0.27	5.94E-03	0.34	4.58E-03	0.56	1.57E-02
	Die3_GM002120	−0.27	5.94E-03	0.34	4.58E-04	0.56	1.57E-02
	Die3_GM002129	−0.53	1.47E-02	0.09	7.56E-03	0.56	7.84E-03
	Die3_GM002539	−0.36	2.36E-03	0.48	9.55E-03	0.79	2.42E-10
	Die3_GM002634	−0.47	8.42E-03	0.50	2.50E-04	0.90	1.22E-14
LysR	Die3_GM000593	−0.16	5.77E-03	0.28	1.18E-02	0.38	9.57E-04
	Die3_GM001419	−0.51	2.15E-02	−0.11	5.66E-03	0.34	7.47E-05
	Die3_GM001928	−0.58	5.23E-04	0.29	9.51E-03	0.82	2.73E-33
	Die3_GM001964	0.47	4.06E-02	0.06	7.30E-03	−0.47	3.31E-05
	Die3_GM002168	−0.56	1.29E-02	0.31	4.02E-02	0.81	8.54E-12
MarR	Die3_GM000785	−0.34	1.42E-03	0.30	6.50E-03	0.57	2.20E-11
	Die3_GM001312	−0.08	8.02E-04	0.20	3.57E-02	0.22	1.39E-01
	Die3_GM001442	−0.53	8.15E-03	0.22	1.06E-02	0.68	3.33E-19
	Die3_GM001826	0.91	1.33E-05	−1.08	1.69E-07	−2.03	2.24E-56

	Die3_GM002346	−0.22	2.59E-02	0.21	7.12E-03	0.38	3.70E-05
	Die3_GM003288	0.20	4.63E-02	−0.02	9.40E-03	−0.28	8.53E-02
	Die3_GM003314	−0.35	1.06E-02	0.15	3.36E-02	0.43	2.55E-07
	Die3_GM003327	−0.29	1.24E-02	0.18	6.31E-03	0.41	2.15E-06
GntR	Die3_GM000046	−0.57	9.15E-03	0.23	1.98E-03	0.73	5.72E-13
	Die3_GM000479	0.51	1.70E-02	−0.94	1.16E-06	−1.49	1.83E-51
	Die3_GM000585	−0.46	1.05E-02	0.12	6.05E-03	0.53	4.39E-04
	Die3_GM001950	0.28	2.29E-02	0.20	1.59E-02	−0.14	2.28E-01
	Die3_GM001987	−0.73	2.71E-04	0.20	6.27E-03	0.87	2.70E-16
	Die3_GM002775	−0.51	7.38E-03	0.28	2.12E-03	0.73	5.70E-15
	Die3_GM003180	−0.34	2.43E-02	0.56	2.56E-03	0.83	3.18E-08
	Die3_GM003271	0.39	7.71E-03	−0.25	1.70E-04	−0.70	1.06E-08
WhiB	Die3_GM000472	−3.08	7.19E-06	−3.89	2.46E-07	−0.83	1.54E-02
	Die3_GM000587	−0.49	3.76E-02	0.39	3.49E-03	0.81	1.52E-15
	Die3_GM000872	0.91	1.64E-03	−2.76	3.90E-05	−3.74	5.95E-21
	Die3_GM002561	−0.38	1.21E-02	0.32	1.85E-03	0.65	1.88E-10
	Die3_GM003069	1.03	2.34E-03	−1.98	6.78E-13	−3.05	1.55E-18
	Die3_GM003322	−2.65	2.23E-17	−2.35	5.98E-11	0.27	1.06E-01
IclR	Die3_GM000996	−0.01	9.84E-03	0.17	2.42E-03	0.12	5.61E-03
	Die3_GM001648	−0.35	2.11E-03	0.25	1.77E-03	0.54	2.33E-04
	Die3_GM001755	−0.58	2.53E-02	0.29	7.20E-03	0.82	2.38E-05
CspA	Die3_GM000278	0.30	4.05E-03	−3.64	3.14E-07	−3.99	5.01E-08
	Die3_GM000500	0.92	1.71E-03	−2.97	7.50E-34	−3.94	4.94E-41
	Die3_GM000721	1.02	8.29E-06	−0.50	1.83E-03	−1.58	5.74E-25
XRE	Die3_GM001282	−0.71	7.02E-04	0.32	3.41E-02	0.97	7.10E-19
	Die3_GM001658	1.55	5.70E-06	−2.24	9.66E-04	−3.83	9.41E-33
	Die3_GM002625	−0.68	5.14E-03	0.34	4.62E-02	0.95	1.51E-15
	Die3_GM002695	0.68	1.75E-02	−1.44	1.83E-08	−2.20	7.10E-42
	Die3_GM002701	−0.72	1.27E-03	0.25	1.30E-03	0.91	1.43E-20
MerR	Die3_GM000265	0.31	1.79E-02	0.11	5.58E-03	−0.26	2.78E-02
	Die3_GM001378	0.96	1.72E-05	−0.78	2.30E-09	−1.80	1.89E-27
	Die3_GM002692	0.69	1.54E-03	0.04	8.62E-03	−0.70	1.90E-08
	Die3_GM002823	0.50	8.37E-03	−0.52	9.08E-03	−1.07	1.10E-06
PadR	Die3_GM000344	−0.48	8.71E-03	0.24	1.95E-03	0.67	7.10E-06
	Die3_GM000350	−0.40	1.69E-04	0.19	4.13E-02	0.53	5.31E-04
	Die3_GM003252	0.35	1.97E-02	−0.22	2.88E-02	−0.62	2.96E-05

The numbers in "C_{16}/Glu", "Pri/Glu" and "Pri/C_{16}" represent the log_2FC values of "C_{16} versus glucose", "pristane versus glucose" and "pristane versus C_{16}". "−" indicates that gene is downregulated.
